# Genetic variation and microRNA targeting of A-to-I RNA editing fine tune human tissue transcriptomes

**DOI:** 10.1186/s13059-021-02287-1

**Published:** 2021-03-09

**Authors:** Eddie Park, Yan Jiang, Lili Hao, Jingyi Hui, Yi Xing

**Affiliations:** 1 Center for Computational and Genomic Medicine, The Children’s Hospital of Philadelphia, Philadelphia, PA 19104 USA; 2 State Key Laboratory of Molecular Biology, Center for Excellence in Molecular Cell Science, Shanghai Institute of Biochemistry and Cell Biology, Chinese Academy of Sciences, Shanghai, 200031 China; 3 National Genomics Data Center & CAS Key Laboratory of Genome Sciences and Information, Beijing Institute of Genomics, Chinese Academy of Sciences, Beijing, 100101 China; 4 Department of Pathology and Laboratory Medicine, University of Pennsylvania, Philadelphia, PA 19104 USA

**Keywords:** A-to-I RNA editing, RNA editing quantitative trait loci, Allele-specific RNA editing, microRNA, Transcript stability, Genetic variation, Single-nucleotide polymorphism, GWAS, RNA-seq, Transcriptome

## Abstract

**Background:**

A-to-I RNA editing diversifies the transcriptome and has multiple downstream functional effects. Genetic variation contributes to RNA editing variability between individuals and has the potential to impact phenotypic variability.

**Results:**

We analyze matched genetic and transcriptomic data in 49 tissues across 437 individuals to identify RNA editing events that are associated with genetic variation. Using an RNA editing quantitative trait loci (edQTL) mapping approach, we identify 3117 unique RNA editing events associated with a cis genetic polymorphism. Fourteen percent of these edQTL events are also associated with genetic variation in their gene expression. A subset of these events are associated with genome-wide association study signals of complex traits or diseases. We determine that tissue-specific levels of ADAR and ADARB1 are able to explain a subset of tissue-specific edQTL events. We find that certain microRNAs are able to differentiate between the edited and unedited isoforms of their targets. Furthermore, microRNAs can generate an expression quantitative trait loci (eQTL) signal from an edQTL locus by microRNA-mediated transcript degradation in an editing-specific manner. By integrative analyses of edQTL, eQTL, and microRNA expression profiles, we computationally discover and experimentally validate edQTL-microRNA pairs for which the microRNA may generate an eQTL signal from an edQTL locus in a tissue-specific manner.

**Conclusions:**

Our work suggests a mechanism in which RNA editing variability can influence the phenotypes of complex traits and diseases by altering the stability and steady-state level of critical RNA molecules.

**Supplementary Information:**

The online version contains supplementary material available at 10.1186/s13059-021-02287-1.

## Background

RNA editing is a cellular process in which the sequence in mature RNA molecules is enzymatically altered from the genomic sequence [1]. The most common type of RNA editing in metazoans is A-to-I RNA editing, the process in which adenosines are deaminated to inosines [2]. RNA editing results in several types of functional consequences such as changes to the protein product of mRNAs, pre-mRNA alternative splicing, transcript localization, and transcript stability [3]. Nonsynonymous coding changes are the most interpretable consequence of RNA editing because inosines are read as guanines by the translation machinery. However, most RNA editing events in humans occur in the noncoding regions of mRNA, such as introns and untranslated regions (UTRs) [4–6]. A-to-I RNA editing is mediated by members of the ADAR (Adenosine Deaminase Acting on RNA) family [2]. In humans, the ADAR family consists of three members: ADAR (ADAR1), ADARB1 (ADAR2), and ADARB2 (ADAR3) [3]. ADAR has a 110 kDa isoform (p110) and a 150 kDa isoform (p150). The p110 isoform is ubiquitously expressed while the p150 isoform is generated from an alternative promoter and is interferon inducible [3]. ADARB1 has a tissue-restricted expression pattern and is highly expressed in the brain [7]. In mammals, ADAR and ADARB1 are essential for life [8–10]. Double-stranded RNA (dsRNA) is required for substrate recognition of ADAR and ADARB1 [3]. In contrast to ADAR and ADARB1, ADARB2 is not known to have editing activity and is thought to play an inhibitory or regulatory role [11, 12]. Mutations in ADAR cause Aicardi–Goutières Syndrome, an autoimmune disorder affecting the brain and skin [13]. Furthermore, altered levels of RNA editing have been associated with cancer as well as various neurological conditions [14–16].

MicroRNAs (miRNAs) are a class of small noncoding RNAs that regulate gene expression by transcript degradation or by translational repression [17]. Since miRNAs are generated from dsRNA intermediates, RNA editing has been shown to regulate various stages of miRNA biogenesis [3, 18]. Furthermore, RNA editing within miRNAs has been described to change the set of targets of the miRNA [19–21]. Similarly, RNA editing of miRNA binding sites within target transcripts has been shown to alter miRNA targeting [22, 23].

Quantitative trait loci (QTL) mapping of molecular traits is a widely used approach to find genetic effects on gene regulation [24]. QTL studies can provide clues to the molecular mechanisms that govern biological processes. For example, expression QTL (eQTL) analysis shows that genetic variants associated with gene expression are often enriched within enhancer and promoter regions, suggesting that these variants may diminish or enhance the binding of transcription factors [25, 26]. RNA editing quantitative trait loci (edQTL) analysis suggests that RNA secondary structure plays an important role in determining the level of RNA editing at particular sites [27, 28].

Genome-wide association studies (GWAS) have been successful in identifying genetic associations with phenotypic traits [29]. However, in the majority of these studies, it is unknown how the genetic variation causally influences the phenotypic trait. Molecular QTL studies can help identify the underlying molecular mechanism that is responsible for the observed phenotype, bridging the knowledge gap in our understanding of how genetic variability results in phenotypic variability [26]. Additionally, statistical approaches have been developed to determine if molecular QTLs share a genetic basis with GWAS traits [30, 31]. These approaches can be used to reduce false-positive associations between multiple traits [32].

Here, we analyzed cis-regulated RNA editing events using an edQTL and allele-specific RNA editing (ASED) approach across 49 tissues and 437 individuals. We find evidence to suggest that tissue-specific ADAR and ADARB1 levels are responsible for many tissue-specific edQTL signals. Many of these edQTL signals are associated with GWAS traits. Surprisingly, we find that many edQTLs also colocalize with their corresponding genes’ steady-state transcript levels. Furthermore, we find evidence to suggest that miRNAs may play a role in linking RNA editing with steady-state transcript levels by targeting the edited or unedited version of the transcripts. We propose a mechanism in which an edQTL can generate an eQTL signal and consequently affect phenotypes by modulating transcript stability in an editing-specific manner.

## Results

### RNA editing levels vary between individuals and across tissues

**Fig. 1:**
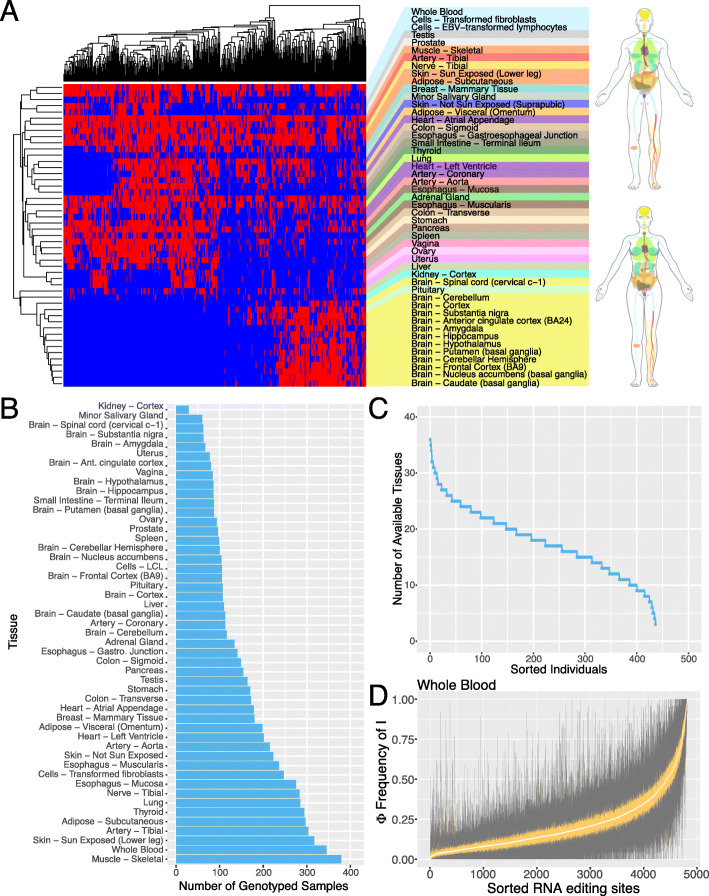
Overview of available data. **a** Heatmap of available datasets. Rows represent tissues and columns represent individuals. Available datasets are in red and unavailable datasets are in blue. Anatomograms on the right are color-coded to correspond to the available tissues. Anatomograms and the color-coding scheme were obtained from the GTEx Portal. **b** Bar plot of the number of available genotyped samples for each analyzed tissue. Tissues are sorted by the number of available genotyped samples. **c** Line plot of the number of available tissues per individual. Individuals are sorted by the number of available tissues. **d** Distribution of RNA editing levels (Φ) within whole blood. Box plots show RNA editing levels of 4815 sites across 345 individuals, with one box plot per site. Sites are sorted by the median Φ value on the *x*-axis. The interquartile ranges for each box plot are represented in orange and the medians are in white. Dark gray lines represent the whiskers of the box plots. Outliers are excluded for clarity

In order to study the factors that influence RNA editing variability across human tissues and individuals, we used genetic and transcriptomic data from the GTEx Project [25]. We analyzed 49 tissues across 437 individuals (Additional file 1: Table S1). To obtain an understanding of the completeness of the data, we generated a heatmap of available datasets in which the tissues and individuals were hierarchically clustered (Fig. 1a). The number of individuals from a given tissue ranges from 29 in kidney to 379 in skeletal muscle (Fig. 1b). The number of tissues from a given individual ranges from 3 to 36 (Fig. 1c). Replicate samples from the same tissue and individual were merged. We restricted our analysis to annotated RNA editing sites [33] and applied a set of filters to focus on RNA editing sites that are expressed and variable between individuals (see the “Methods” section). We observed that RNA editing can be variable across different RNA editing sites and between different individuals (Fig. 1d). Furthermore, RNA editing has a characteristic frequency distribution in which most RNA editing sites are edited less than 50%. However, the range spans from 0 to 100% across all observed tissues (Additional file 2: Figure S1).

### edQTL and ASED analysis identifies cis-regulated RNA editing events across human tissues

**Fig. 2:**
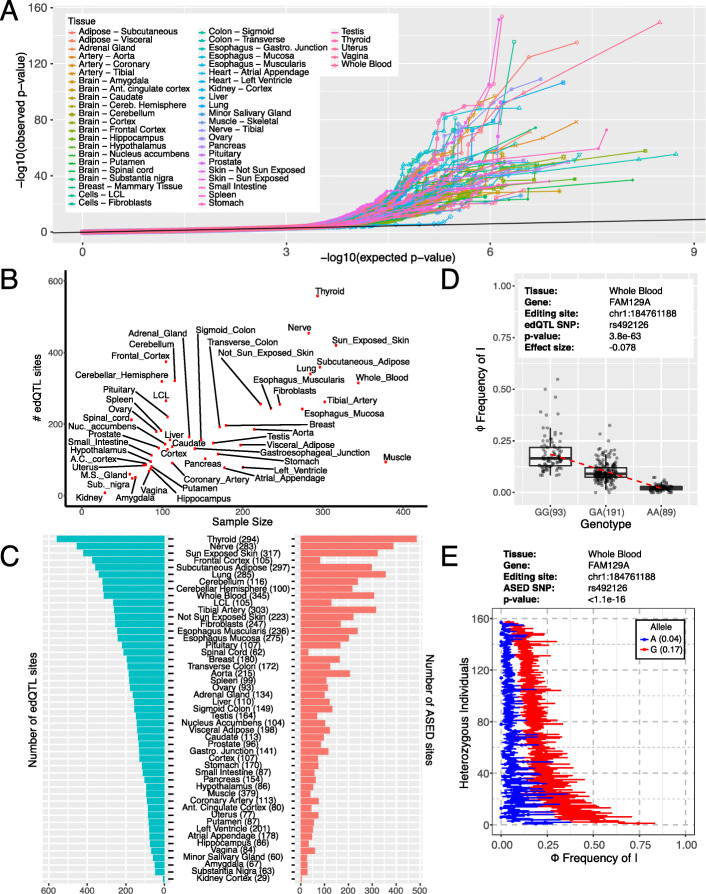
Cis variation of RNA editing identified by edQTL and ASED analysis. **a** Quantile-quantile plot (qq-plot) testing association of RNA editing levels with cis genetic polymorphisms across 49 tissues. Black line indicates values for which the observed *p* value is equal to the expected p-value. **b** Scatter plot of the number of edQTL sites vs sample size across the 49 tissues. **c** Histograms of the number of edQTL sites (left) and ASED sites (right) across all tissues. Tissues are sorted by the number of edQTL sites. **d** Example of an edQTL site in the FAM129A gene. Box plots show the significant association of rs492126 with the editing level (Φ) at chr1:184761188 within the whole blood. Each dot represents data from a particular individual. The dashed red line represents a linear fit of the data. **e** Example of allele-specific RNA editing in the FAM129A gene. ASED analysis identifies RNA editing site chr1:184761188 with respect to heterozygous SNP rs492126. For each heterozygous individual (*y*-axis), blue and red points indicate editing levels for each allele (*x*-axis). Error bars represent likelihood-ratio test-based 95% confidence intervals of RNA editing levels inferred from allele-specific read counts. Average allelic Φ values are shown in parentheses

We used an edQTL approach as the primary means to identify cis-regulated RNA editing events and found 3117 unique RNA editing sites that are associated with at least one edQTL SNP across 49 tissues (Additional file 3: Table S2). Across all tissues, we found RNA editing sites associated with genetic variants (Fig. 2a). The number of edQTL sites per tissue range from 8 in kidney to 558 in thyroid (Fig. 2b). Across most tissues, we observed a correlation between the number of edQTL sites detected and the sample size (i.e., the number of genotyped individuals for that tissue). However, skeletal muscle has fewer than expected number of edQTL sites relative to its large sample size, and certain neuronal-related tissues such as frontal cortex have greater than expected number of edQTL sites (Fig. 2b). After normalizing for the number of tested sites, we observed a strong linear relationship (Additional file 2: Figure S2), suggesting that more edQTL signals can be detected with a larger sample size or deeper sequencing, and that the fewer than expected number of edQTL sites detected in skeletal muscle is due to the depletion of RNA editing events in that tissue.

To complement our edQTL analysis, we also performed an ASED analysis to identify 1986 unique allele-specific RNA editing sites (Additional file 4: Table S3). Together, we found 4347 unique sites in the union of the edQTL and ASED analysis (Fig. 2c). Furthermore, edQTL/ASED sites are often shared between tissues (Additional file 2: Figure S3). An example of an RNA editing site that is strongly associated with a genetic polymorphism is seen in the 3′-UTR of FAM129A (Fig. 2d). Here, a higher level of RNA editing at chr1:184761188 is associated with the G-allele of rs492126 while a lower level of RNA editing is associated with the A-allele. The median RNA editing levels for GG, GA, and AA genotypes are 0.17, 0.09, and 0.02, respectively. Similarly, in the ASED analysis, the G-allele has a median editing level of 0.17 and the A-allele has a median editing level of 0.04, consistent with the edQTL analysis (Fig. 2e).

### Tissue-specific edQTL signals are influenced by ADAR and ADARB1 levels

**Fig. 3:**
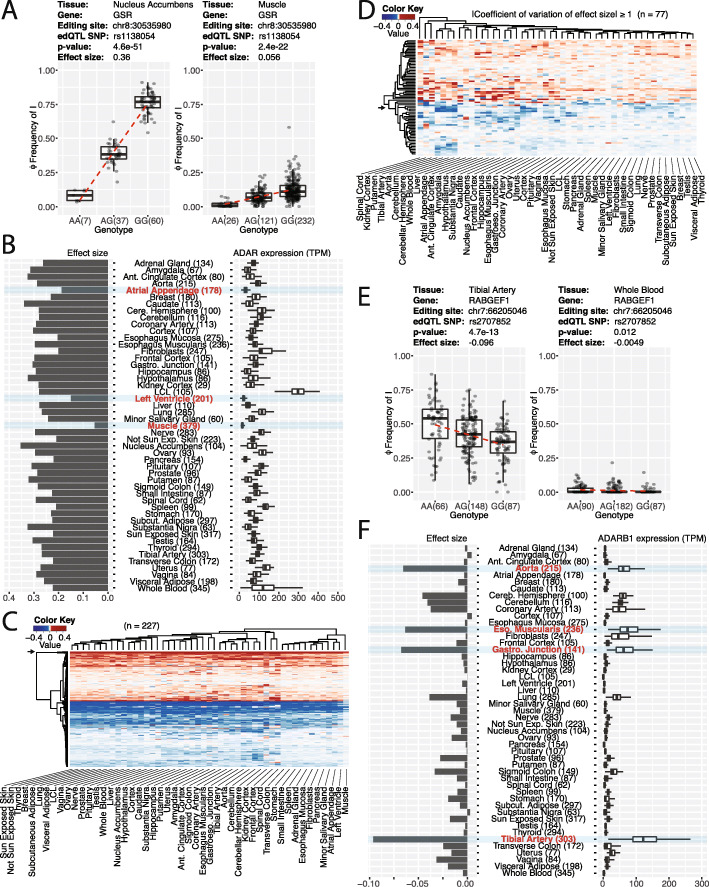
Tissue-specific edQTL signals. **a** Example of a tissue-specific edQTL site with small effect size in skeletal muscle. Box plots show the association of rs1138054 with the editing level (Φ) at chr8:30535980 in GSR within the nucleus accumbens (left) and skeletal muscle (right). Each dot represents data from a particular individual. The dashed red line represents a linear fit of the data. **b** Low ADAR expression level in skeletal muscle correlates with small edQTL effect size. Bar plot (left) shows effect sizes of the association of rs1138054 with the editing level (Φ) at chr8:30535980 in GSR across 49 tissues. Box plot (right) shows ADAR expression level across 49 tissues, with outliers removed for clarity. Three tissues with smallest effect size and lowest ADAR expression level are highlighted. **c** Heatmap of edQTL effect sizes for 227 RNA editing sites (rows) across 49 tissues (columns). Only RNA editing sites with sufficient coverage to pass filters and have effect sizes computed across all 49 tissues are included in the plot. The RNA editing site in GSR as described in **a** and **b** is indicated with the arrow. **d** Heatmap of edQTL effect sizes for 77 RNA editing sites (rows) with large variation in edQTL effect sizes (|coefficient of variation| ≥ 1) across 49 tissues (columns). Only RNA editing sites with sufficient coverage to pass filters and have effect sizes computed across all 49 tissues are included in the plot. The RNA editing site in RABGEF1 as described in **e** and **f** is indicated with the arrow. **e** Example of a tissue-specific edQTL site with small effect size in whole blood. Box plots show the association of rs2707852 with the editing level (Φ) at chr7:66205046 in RABGEF1 within the tibial artery (left) and whole blood (right). Each dot represents data from a particular individual. The dashed red line represents a linear fit of the data. **f** Tissues with high ADARB1 expression level have a tissue-specific set of edQTL sites. Bar plot (left) shows effect sizes of the association of rs2707852 with the editing level (Φ) at chr7:66205046 in RABGEF1 across 49 tissues. Box plot (right) shows ADARB1 expression level across 49 tissues, with outliers removed for clarity. Tissues with largest effect size and highest ADARB1 expression level are highlighted

We compared the effect sizes of edQTLs across 49 tissues. We observed that edQTL sites in skeletal muscle tend to have smaller effect sizes than other tissues. For example, the edQTL site at chr8:30535980 with respect to rs1138054 in the glutathione reductase (GSR) gene has a large effect size of 0.36 in the nucleus accumbens but a small effect size of 0.056 in skeletal muscle (Fig. 3a). This edQTL site is in an inverted ALU hairpin in the 3′-UTR of GSR (Additional file 2: Figure S4A). GSR is responsible for maintaining the cellular level of glutathione, an important antioxidant to prevent damage from reactive oxygen species [34]. We chose to investigate this site because GSR is ubiquitously expressed and the edQTL has a large effect size in most tissues except for skeletal muscle. Furthermore, the change in RNA editing appears to be driven by a change in the RNA secondary structure in which the edQTL SNP alters the base-pairing at the RNA editing site across the inverted ALU hairpin (Additional file 2: Figure S4B). To comprehensively investigate the effects of edQTLs on computationally predicted RNA secondary structure, we adopted the approach from [27]. We found that edQTL SNPs are closer to their corresponding RNA editing sites, have a larger impact on the number of paired bases, and have a greater effect on the minimum free energy of the predicted RNA secondary structure, compared to control SNPs (Additional file 2: Figure S4C, D, E). These results are consistent with our prior observation on a much smaller set of edQTL sites in a single cell type [27].

The effect size for the GSR edQTL site (chr8:30535980) is relatively consistent across all tissues but much smaller in skeletal muscle. Coincidentally, skeletal muscle has the lowest expression level of ADAR (Fig. 3b). This trend is consistent across the 227 edQTL sites that have a measurable effect size across all 49 tissues, with skeletal muscle being the tissue with the lowest edQTL effect size (Fig. 3c). To more formally assess if the genotype effect on a given RNA editing site is tissue-dependent (muscle vs non-muscle), we applied a multivariate model and likelihood ratio test to the 10 edQTL sites with the largest average genotype effect size across 49 tissues among the 227 sites used to generate Fig. 3c (i.e., edQTL sites with sufficient RNA-seq coverage and measurable effect sizes in all 49 tissues). We chose to focus on the top 10 edQTL sites to obtain a high signal-to-noise ratio in the estimated effect sizes, so we can reliably dissect genotype and tissue effects on RNA editing levels. For all 10 sites, the likelihood ratio test for the genotype x tissue interaction term is highly significant (Additional file 2: Figure S5A), suggesting that the genotype effect on RNA editing levels is tissue-dependent and differs significantly between muscle vs non-muscle tissues. Based on the estimated effect sizes in muscle vs non-muscle tissues, we found that muscle typically has an 80% reduction in genotype effect size compared to non-muscle tissues (Additional file 2: Figure S5B-C). Interestingly, the two heart tissues (atrial appendage, left ventricle) also have low levels of ADAR and smaller edQTL effect sizes. Based on these observations, we reasoned that the low ADAR level in skeletal muscle contributes to a global reduction in the effect size of edQTL signals.

For most edQTL sites, the effect sizes are comparable across all tissues (with the exception of muscle). However, for a small fraction of edQTL sites, some additional tissues (other than muscle) also have varying effect sizes. After inspecting RNA editing sites with a high variance of effect sizes (|coefficient of variation| ≥ 1) across tissues (Fig. 3d), we identified a number of clusters in which the effect size has a non-uniform distribution across tissues. An example of an RNA editing site that demonstrates this type of tissue specificity is in RABGEF1 at chr7:66205046 (Fig. 3e). RNA editing is significantly associated with rs2707852 in some tissues but not in others. We found that the tissues with higher effect sizes at this site tend to express higher levels of ADARB1 (Fig. 3f). There are 11 edQTL sites that have effect sizes correlated (*R*^2^ ≥ 0.5) with ADARB1 levels (Additional file 5: Table S4). This suggests that the presence of ADARB1 is responsible for generating tissue-specific edQTL signals in these tissues.

### Cis-regulated RNA editing and miR-125a-3p fine-tune steady-state transcript levels of DHFR

Next, we aimed to identify edQTL sites that are known to be associated with human disease. We intersected our sites with the Editome Disease Knowledgebase [35] and found a study that showed RNA editing at chr5:79923430 is responsible for upregulating dihydrofolate reductase (DHFR) in breast cancer [36]. Specifically, Nakano et al. [36] showed that miR-125a-3p targets the unedited transcripts of DHFR and results in reduced mRNA and protein levels. ADAR-mediated RNA editing at chr5:79923430 reduces miRNA targeting of the transcripts, which results in the upregulation of DHFR. Increased levels of DHFR in breast cancer result in enhanced cellular proliferation and resistance to methotrexate, a chemotherapy agent and immune system suppressant.

**Fig. 4:**
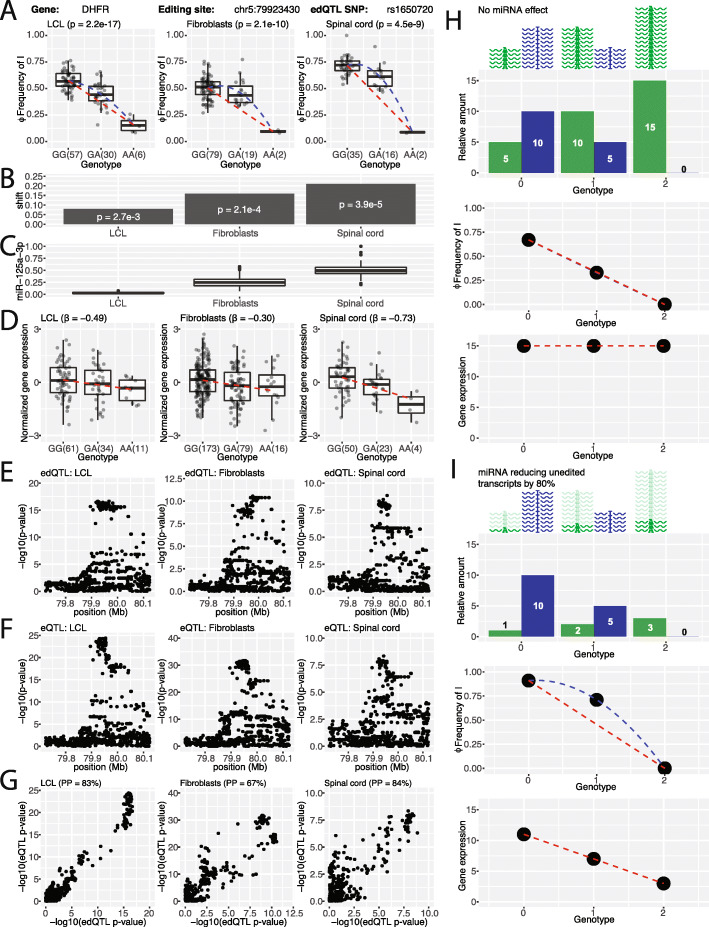
Steady-state transcript levels regulated by RNA editing and miRNA mediated transcript degradation. **a** edQTL signals in the DHFR gene for LCL, fibroblasts, and spinal cord. Box plots show the significant association of rs1650720 with the editing level (Φ) at chr5:79923430. Each dot represents data from a particular individual. edQTL *p* values are shown in parentheses. The dashed blue curve represents a quadratic fit of the data while the dashed red line represents a linear fit of the homozygous individuals. **b** Non-linearity of edQTL signals measured by the difference between the centers of the quadratic fit (dashed blue curve) and the linear fit using the homozygous individuals (dashed red line). **c** Box plots of the relative levels of miR-125a-3p inferred from RNA-seq data. **d** DHFR eQTL signal. Box plots show the significant association of rs1650720 with the normalized DHFR gene expression level. Each dot represents data from a particular individual. The dashed red line represents a linear fit of the data. **e**, **f** Manhattan plots showing the −log10(*p* value) for RNA editing edQTL (**e**) and gene expression eQTL (**f**) in a 400-kb window centered at the RNA editing site. **g** Scatter plot of −log10(*p* value) from edQTL and eQTL signals suggests colocalization of RNA editing variation and gene expression variation. Colocalization posterior probabilities are shown in parentheses. **h**, **i** Simulation of edQTL and eQTL signals with no miRNA effect on transcript degradation (**h**) and with an 80% miRNA effect on degradation of unedited transcripts (**i**). A schematic illustration and corresponding bar plot show the simulated levels of unedited (green) and edited (blue) transcripts across three genotypes (top). Simulated RNA editing levels and gene expression levels across three genotypes are plotted (bottom). In the edQTL plots, the dashed blue curve represents a quadratic fit of the data while the dashed red line represents a linear fit of the homozygous individuals. In the eQTL plots, the dashed red line represents a linear fit of the data

In our edQTL analysis, the RNA editing site at chr5:79923430 is significantly associated with rs1650720 in three tissues: lymphoblastoid cell lines (LCL), fibroblasts, and spinal cord (Fig. 4a). Interestingly, there are deviations from linearity when we examined the correlation between genotypes and RNA editing levels in these three tissues (Fig. 4b). Specifically, the RNA editing levels that we observed for the heterozygous individuals differ from the expected values, based on the observed levels for the homozygous individuals. Based on the work of Nakano et al. [36], we reasoned that the nonlinearity is due to the effect of the miRNA preferentially targeting the unedited transcripts. Indeed, the degrees of nonlinearity correlate with miR-125a-3p levels (Fig. 4c). These results also suggest that an edQTL signal would result in an eQTL signal in the presence of this miRNA. As the RNA editing levels change across different genotypes, the level of miRNA-mediated transcript degradation would also change across different genotypes. Indeed, an eQTL signal was also observed for DHFR with respect to rs1650720, with the strongest eQTL signal observed in spinal cord (Fig. 4d), where miR-125a-3p has the highest expression level among the three tissues (Fig. 4c) and the non-linearity of the edQTL signal is also the strongest (Fig. 4a and b). Furthermore, allele-specific expression (ASE) analysis confirms higher expression of the reference allele in all three tissues (Additional file 2: Figure S6). We should note that in these analyses, the expression levels of miR-125a-3p in the three tissues were estimated via a proxy approach, based on GTEx poly-A selected RNA-seq reads that align to the cleavage product of the primary miRNA transcript. This proxy approach has a precedent in the literature. A similar approach has been evaluated and used by the FANTOM consortium to estimate mature miRNA levels using CAGE (an approach to measure the 5′ ends of RNA molecules) [37]. To assess the validity of the proxy method we employed in this work, we compared this method as applied to GTEx tissues with a published miRNA qPCR dataset of human tissues [38], on a set of 20 tissues shared between the two datasets. We compared miRNA quantifications of four well-characterized tissue-specific miRNAs (brain-specific miR-9, brain-specific miR-124a, liver-specific miR-122a, muscle-specific miR-1), as well as miR-125a. For the four tissue-specific miRNAs, the proxy method performs as well as qPCR in identifying their tissue specificity (Additional file 2: Figure S7A, B, C, D). For miR-125a, the miRNA levels estimated by the proxy method and by qPCR had a Spearman correlation of 0.55 (Additional file 2: Figure S7E), even though the tissue materials and sampling sites were not identical between the two datasets. Taken together, these data suggest that this proxy approach provides a reasonable approximate estimate of mature miRNA levels.

We tested whether there may be a shared causal variant for RNA editing levels at chr5:79923430 and DHFR transcript levels using colocalization analysis. Stacked Manhattan plots for edQTL (Fig. 4e) and eQTL (Fig. 4f) signals suggest a colocalization of association signals. We applied a Bayesian test for colocalization [30] and created scatter plots (Fig. 4g) comparing these signals. The posterior probability of colocalization of edQTL and eQTL signals in LCL, fibroblasts, and spinal cord is 83%, 67%, and 84%, respectively. This suggests that there is a shared genetic basis that influences the RNA editing levels at chr5:79923430 and the steady-state transcript levels of DHFR. Taken together, these results support a model in which RNA editing can affect steady-state transcript levels by modulating miRNA targeting. Furthermore, edQTL signals can give rise to eQTL signals through this mechanism.

To further support this model, we performed simulation studies based on two scenarios: one in which there is no miRNA degradation effect (Fig. 4h) and the other in which a miRNA reduces the level of the unedited transcripts by 80% (Fig. 4i). When there is no miRNA degradation effect, our simulation suggests that the edQTL signal is linear while the eQTL signal is flat (i.e., no association between genotype and steady-state transcript level) (Fig. 4h). When miRNA degrades the unedited transcripts, the edQTL signal becomes non-linear because of an imbalance in steady-state levels of unedited vs edited transcripts, and an eQTL signal also emerges (Fig. 4i). This preferential degradation may account for the smaller than expected sample size of the AA genotype in Fig. 4a, as certain samples were excluded from the analysis and plot due to low RNA-seq coverage of the RNA editing site. We investigated additional scenarios with varying simulation parameters such as the initial edQTL effect size and miRNA-mediated degradation rate (Additional file 2: Figure S8), and if the miRNA targets the edited transcripts (Additional file 2: Figure S9). We obtained similar results.

### Cis-regulated RNA editing is associated with complex traits

We aimed to investigate phenotypic consequences that result from these edQTL events. Our first approach was to identify edQTL sites that create nonsynonymous changes within protein-coding regions. We found 15 sites that create a nonsynonymous amino acid change (Additional file 6: Table S5). For one of these sites, the functional impact of RNA editing had been previously characterized [39]. Specifically, RNA editing in NEIL1 at chr15:75646086 results in a K242R change that alters the enzymatic property of the DNA repair enzyme [39]. Our analysis shows that RNA editing at this site is associated with SNP rs34879829. Individuals with the C-allele have higher RNA editing levels at this site while individuals with the T-allele have lower RNA editing levels (Additional file 2: Figure S10). This suggests a possible genetic mechanism in which altered DNA repair activity could result in an accumulation of mutations and an increased predisposition to cancer.

**Fig. 5:**
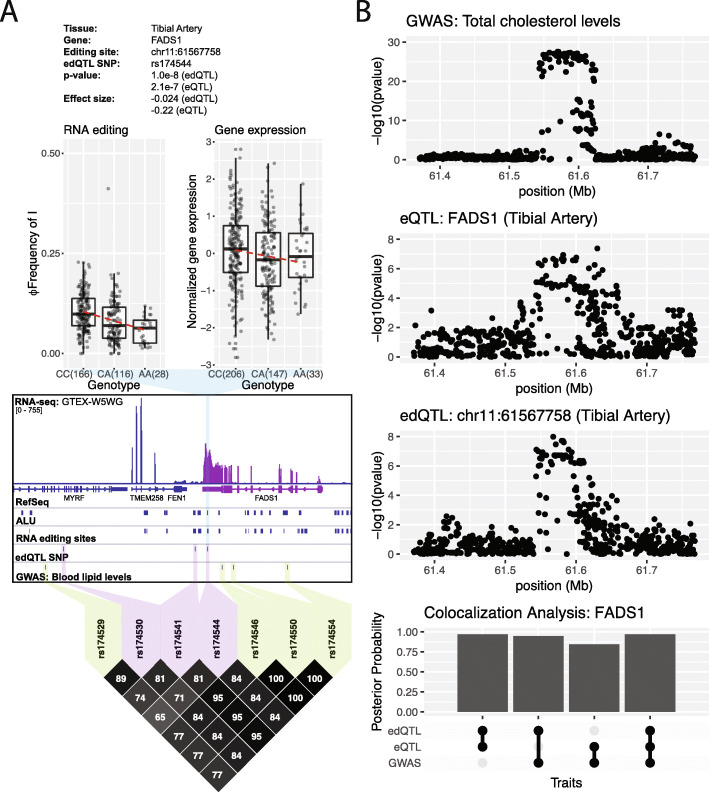
Colocalization analysis between edQTL, eQTL, and GWAS signals of the FADS1 gene. **a** Box plots show the significant association of rs174544 with the editing level (Φ) at chr11:61567758 and gene expression level of the FADS1 gene within the tibial artery (top). Each dot represents data from a particular individual. An example of a tibial artery RNA-seq alignment is shown along with gene annotations (RefSeq), annotated ALU elements, annotated RNA editing sites, edQTL SNPs for chr11:61567758, and GWAS SNPs (middle). LD plot (bottom) shows GWAS SNPs (green) linked with edQTL SNPs (purple) in FADS1. For clarity, the GWAS traits (HDL, LDL, total cholesterol, and triglycerides) identified by Hoffmann et al. [40] are displayed. **b** edQTL and eQTL signals of the FADS1 gene colocalize with GWAS signals for blood lipid levels. Manhattan plots for total cholesterol, gene expression, and RNA editing are shown (top). Bar plot shows colocalization posterior probabilities between edQTL, eQTL, and GWAS signals (bottom)

Of the 3117 edQTL sites, 700 (Additional file 7: Table S6) are associated with 461 GWAS traits (LD *R*^2^ > 0.8). Four hundred forty-three RNA editing sites (Additional file 8: Table S7) colocalize with the expression level of their respective genes (posterior probability > 0.75) in at least one tissue. One hundred thirty-one RNA editing sites are in both groups. One of the RNA editing sites at the intersection of these two groups is the RNA editing site at chr11:61567758 in the 3′-UTR of FADS1 (Fig. 5a). FADS1 is a member of the fatty acid desaturase (FADS) gene family which encodes enzymes that are responsible for the biosynthesis of long chain polyunsaturated fatty acids (LCPUFAs) [41]. This RNA editing site is linked with 81 unique GWAS traits from 67 GWAS publications (Additional file 2: Figure S11). Most of these GWAS traits are related to various blood lipid levels and metabolic traits. This locus is located in an 85 kb LD block within the FADS gene cluster. Changes in the diet within a population have been shown to modulate the direction of adaptation for this locus [42]. Alleles limiting LCPUFA biosynthesis were adaptive in European populations prior to the advent of farming when diets were rich in fish and meat (LCPUFAs-rich diets) while alleles enhancing LCPUFA biosynthesis were adaptive after the advent of farming when diets were more plant-based (LCPUFAs-poor diets) [42]. Similarly, this trend is observed in modern human populations in which LCPUFA levels within regional diets correlate with these alleles. For instance, alleles favoring LCPUFA biogenesis are enriched in south Asian populations whose diets are largely plant based [42]. By contrast, alleles limiting LCPUFA biogenesis are enriched in Eskimo populations whose diets are rich in fish and meat [42].

We found that RNA editing levels at chr11:61567758 and FADS1 transcript levels correlate with SNP rs174544. The C-allele is associated with higher RNA editing levels, higher FADS1 transcript levels, and higher blood lipid levels. Conversely, the A-allele is associated with lower RNA editing levels, lower FADS1 transcript levels, and lower blood lipid levels (Fig. 5a). Given that FADS1 is a key factor in the biogenesis of LCPUFAs, FADS1 expression is likely upstream of various GWAS traits related to blood lipid levels. We hypothesized that RNA editing may modulate steady-state FADS1 transcript levels, similar to the DHFR case. Indeed, pairwise and multiple-trait colocalization analysis between edQTL, eQTL, and GWAS signals suggests that there may be a shared genetic factor underlying these traits (Fig. 5b). Taken together, these results suggest that RNA editing may play a role in linking genetic variation with phenotypic GWAS traits by modulating the transcript levels of FADS1. However, it is unclear whether this is through miRNA-mediated degradation or another mechanism.

### RNA editing and miRNAs interact to alter steady-state transcript levels and complex traits

**Fig. 6:**
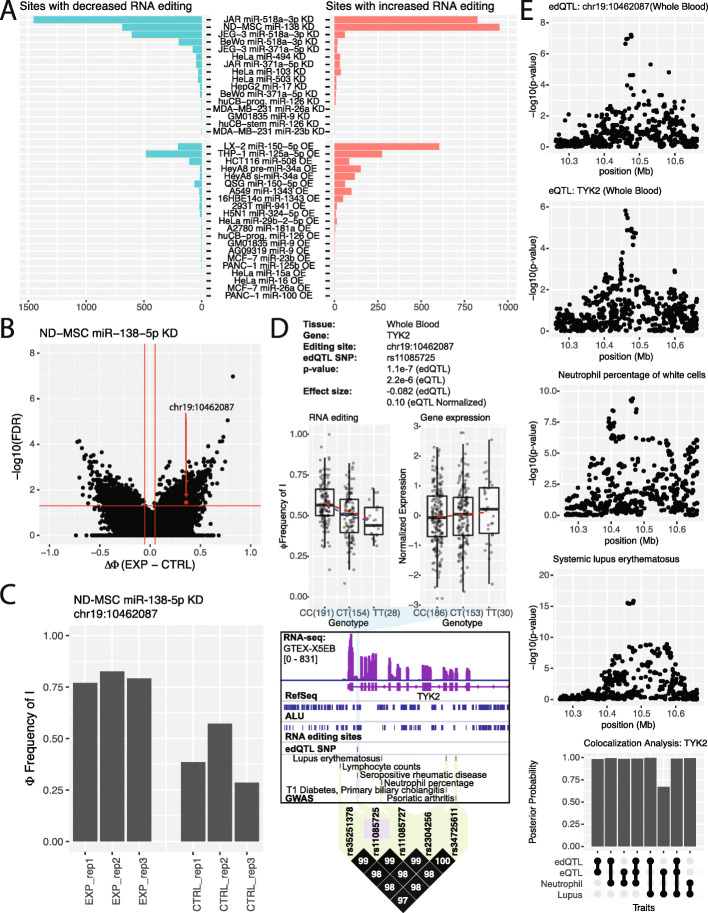
Identification of miRNAs linking edQTL, eQTL, and GWAS signals. **a** Histograms of the number of differential RNA editing sites induced by miRNA perturbation. The numbers of sites with a significant decrease in RNA editing level are plotted on the left while the numbers of sites with a significant increase in RNA editing level are plotted on the right. The vertical axis labels indicate the cell line, miRNA, and the type of perturbation (KD, knockdown; OE, overexpression). **b** Example of differential RNA editing sites induced by miRNA perturbation (miR-138-5p knockdown in ND-MSC cells). Horizontal red line indicates 5% FDR. Two vertical red lines indicate a change in RNA editing level of − 5% and 5%. The red dot represents the RNA editing site at chr19:10462087 in the TYK2 gene. **c** RNA editing level at chr19:10462087 in the TYK2 gene upon miR-138 knockdown in ND-MSC cells. **d** Cis-regulated RNA editing at chr19:10462087 is associated with cis-regulated gene expression of the TYK2 gene and immune system related GWAS traits. Box plots show the significant association of rs11085725 with the editing level (Φ) at chr19:10462087 and gene expression level of the TYK2 gene within the whole blood (top). Each dot represents data from a particular individual. An example of a whole blood RNA-seq alignment is shown along with gene annotations (RefSeq), annotated ALU elements, annotated RNA editing sites, edQTL SNPs for chr19:10462087, and GWAS SNPs (middle). LD plot (bottom) shows GWAS SNPs (green) linked with the edQTL SNP (purple) in TYK2. **e** edQTL and eQTL signals of the TYK2 gene colocalize with GWAS signals for neutrophil percentage of white blood cells and systemic lupus erythematosus. Manhattan plots for RNA editing, gene expression, and two GWAS traits are shown (top). Bar plot shows colocalization posterior probabilities between edQTL, eQTL, and GWAS signals (bottom)

In order to find additional examples of cis-regulated RNA editing events that modulate transcript levels through miRNA-mediated degradation, we first sought to identify RNA editing sites that have the potential to regulate miRNA targeting. We obtained RNA-seq data from 48 miRNA perturbation (overexpression or knockdown) experiments across 29 cell lines and 27 miRNAs (Additional file 9: Table S8). We looked for RNA editing sites that change when a miRNA is perturbed. Most of these miRNA perturbation experiments had little to no effect on RNA editing. However, a subset of these miRNA perturbations resulted in significant changes to RNA editing (Fig. 6a). Through this analysis, we were able to identify 7293 RNA editing sites that are potential targets of a miRNA (Additional file 10: Table S9), 245 of which are also edQTL sites. Sixteen of these were associated with both a GWAS trait and transcript levels of their respective genes.

One of these 16 sites is the RNA editing site chr19:10462087 in the TYK2 gene. This site was significantly altered upon miR-138-5p knockdown (Fig. 6b, c). Downregulation of miR-138-5p resulted in significantly increased RNA editing at this site. This suggests that miR-138-5p may preferentially target the edited version of TYK2 over the unedited version. If a miRNA targets the edited version of this transcript, simulations predict that the edQTL and eQTL would have opposite directions (Additional file 2: Figure S9), which agrees with our observations (Fig. 6d). Specifically, as RNA editing at chr19:10462087 decreases across the genotypes of rs11085725 (from CC to CT to TT), the level of miRNA-mediated degradation decreases and the steady-state transcript level increases. This locus is in LD with several GWAS traits related to immune function and disease. Two of these GWAS traits (neutrophil percentage of white blood cells [43] and systemic lupus erythematosus [44]) have available summary statistics and colocalize with the edQTL and eQTL signals (Fig. 6e). Furthermore, these two GWAS traits colocalize with each other. Taken together, these data suggest that an edQTL signal in conjunction with miR-138-5p induces an eQTL for TYK2 and consequently impacts downstream phenotypic traits. Furthermore, given the colocalization of the two GWAS traits and their functional similarities, variants in this locus may alter neutrophil levels and subsequently the development of systemic lupus erythematosus, or vice versa. However, based on the evidence collected, we do not know whether miR-138-5p interacts with this RNA editing site in TYK2 directly (via a direct miRNA-mRNA interaction), or indirectly (via miRNA regulation of another trans-acting regulator such as an RNA binding protein).

### Direct edQTL-miRNA interactions mediate tissue-specific edQTL:eQTL colocalization events

**Fig. 7:**
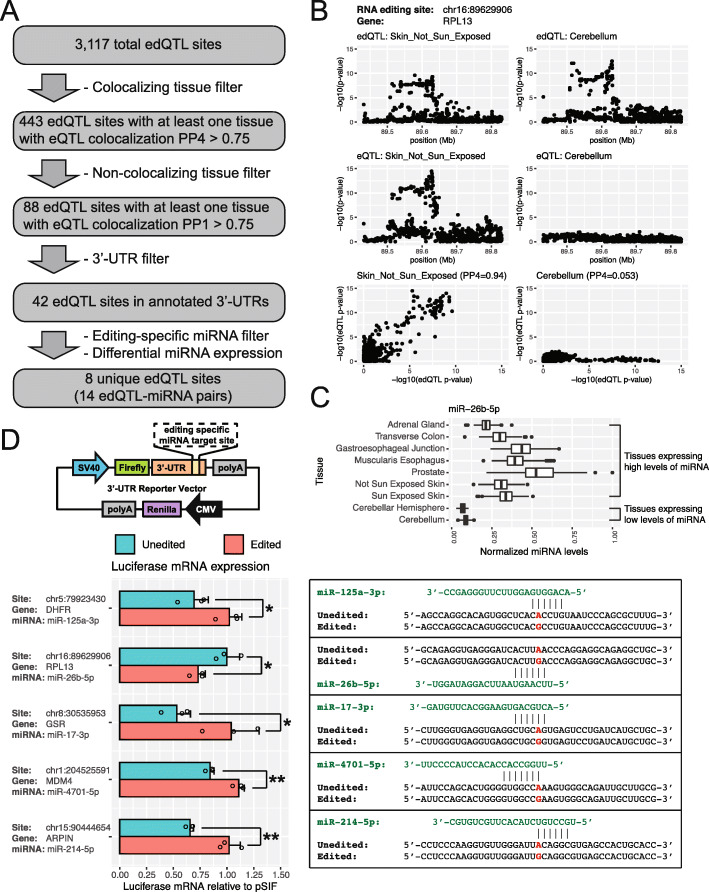
Computational discovery and experimental validation of miRNA-mediated tissue-specific edQTL:eQTL colocalization events. **a** Flowchart of computational analysis. Each edQTL site was required to have at least one tissue with colocalizing edQTL and eQTL signals (PP4 > 0.75) and at least one tissue with non-colocalizing edQTL and eQTL signals (PP1 > 0.75), be in the 3′-UTR, and have an editing-specific miRNA which is differentially expressed (fold change > 2) between the colocalizing and non-colocalizing tissues. **b** Example of an edQTL event in RPL13 with colocalizing edQTL and eQTL signals in the skin (not sun exposed) (left column) and non-colocalizing edQTL and eQTL signals in the cerebellum (right column). Manhattan plots for edQTL (top row), eQTL (middle row), and scatter plots of −log10(*p* value) from edQTL and eQTL signals (bottom row) show the presence (left column) or absence (right column) of edQTL:eQTL colocalization. Colocalization posterior probabilities are shown in parentheses (bottom row). **c** Differential expression of an editing-specific miRNA (miR-26b-5p) targeting the edited version of RPL13. Tissues expressing high levels of the miRNA have colocalizing edQTL and eQTL signals. Tissues expressing low levels of the miRNA do not have colocalizing edQTL and eQTL signals. **d** Experimental validation of miRNA-mediated edQTL:eQTL colocalization events. Diagram of the 3′-UTR reporter vector using plasmids containing 3′-UTR fragments with the edQTL RNA editing sites (top). Luciferase mRNA levels measured by qPCR of tested 3′-UTR constructs in the presence of editing-specific miRNAs (bottom left). Each barplot displays the mean and SD of three independent experiments. *p < 0.05, **p < 0.01. Diagram indicating the editing-specific targeting of miRNAs to unedited or edited 3′-UTR sequences (bottom right). Guanine was used in place of inosine to indicate an edited site

We carried out computational and experimental analyses to investigate if miRNAs can mediate edQTL:eQTL colocalization via direct miRNA-mRNA interactions. Specifically, we performed an integrative computational analysis of edQTL, eQTL, and miRNA expression profiles across diverse tissues, as well as miRNA sequence complementarity to RNA editing sites, to predict edQTL-miRNA pairs for which the miRNA may generate an eQTL signal from an edQTL locus in a tissue-specific manner (Fig. 7a). Starting from all 3117 edQTL sites, we performed edQTL:eQTL colocalization analyses tissue-by-tissue and identified 88 edQTL sites with tissue-specific colocalization (i.e., strong evidence of colocalization in at least one tissue and non-colocalization in at least one tissue). Focusing on 42 such edQTL sites located in annotated 3′-UTRs, we used TargetScan [45] to identify miRNAs that may specifically target the edited or unedited version of the mRNA and used the GTEx RNA-seq data to discover tissue-specific miRNAs (see the “Methods” section). By intersecting the TargetScan and GTEx results, we identified 8 unique edQTL sites involving 14 edQTL-miRNA pairs (Fig. 7a and Additional file 11: Table S10). Each candidate edQTL site was required to have at least one tissue with colocalizing edQTL and eQTL signals (PP4 > 0.75) and at least one tissue with non-colocalizing edQTL and eQTL signals (PP1 > 0.75), be in the 3′-UTR, and have an editing-specific miRNA which is differentially expressed (fold change > 2) between the colocalizing and non-colocalizing tissues. An example involving an edQTL site in RPL13 and miR-26b-5p is illustrated in Fig. 7b, c. For this edQTL site, a signal for edQTL:eQTL colocalization is present in the skin while the colocalization signal is absent in the cerebellum (Fig. 7b). Furthermore, the predicted miRNA (miR-26b-5p) is expressed at substantially higher levels in the colocalizing tissues compared to the non-colocalizing tissues (Fig. 7c) and specifically targets the edited version of the transcript (Fig. 7d).

In order to test the validity of this analysis, we performed 3′-UTR luciferase reporter assays to test the effect of RNA editing on RNA stability in the presence of the miRNA. We tested four of the predicted edQTL sites as well as the DHFR site as a positive control (Fig. 7d). For each RNA editing site, we co-transfected the edited or unedited version of the 3′-UTR reporter with the predicted miRNA, with guanine used in place of inosine to model the effect of RNA editing. For all five sites tested, the experimental data show that the miRNA of interest specifically targets the unedited or edited version of the 3′-UTR for transcript degradation, in a manner consistent with our computational prediction based on the edQTL and eQTL signals. Specifically, four of the five tested sites (including the positive-control DHFR site) have a miRNA targeting the unedited transcript. For these sites, the unedited version of the reporter had significantly lower mRNA levels compared to the edited version when co-transfected with the miRNA. Conversely, for the tested site in RPL13 that has a miRNA targeting the edited transcript, the edited version of the reporter had significantly lower mRNA levels compared to the unedited version when co-transfected with the miRNA. We obtained comparable results at the protein level (Additional file 2: Figure S12). Taken together, these results indicate that our proposed mechanism for edQTL:eQTL colocalization generalizes beyond the DHFR example to other genes and RNA editing sites.

## Discussion

A-to-I RNA editing is widespread in human transcriptomes and influences multiple layers of gene regulation [3, 46]. Recent studies have used population-scale RNA-seq data to survey the genetic variation of RNA editing in selected cell types or tissues [27, 47, 48]. In this work, using matched genetic and transcriptomic data in 49 tissues across 437 human individuals, we sought to delineate the comprehensive landscape and investigate the tissue specificity of genetically regulated RNA editing events. Using two complementary analytic approaches, we identified 3117 edQTL RNA editing sites and 1986 allele-specific RNA editing sites across 49 human tissues, including 756 sites that were identified by both approaches. We also found that the edQTL signals of specific RNA editing sites could vary across tissues. For example, we found a set of tissue-specific edQTL sites, whose variation in edQTL effect sizes across tissues is correlated with ADARB1 expression level (Fig. 3f, Additional file 5: Table S4). We also observed generally weaker edQTL signals in skeletal muscle, consistent with low ADAR expression level in this tissue [49]. These tissue-specific variations in edQTL signals may be attributed to differences in baseline RNA editing levels, as dependent on the concentrations of RNA editing enzymes (ADAR or ADARB1) in a given tissue. We have compiled our results into an easy-to-use web server for readers to explore the data (https://xingshiny.research.chop.edu/edqtl/).

Colocalization analysis has become a widely used approach to find associations between molecular and/or phenotypic traits by comparing the overlap between their association signals [30, 32]. We found 443 edQTL sites for which the edQTL signals colocalize with the eQTL signals of their respective genes in at least one tissue. We should note that the colocalization analysis is not based on the genomic distance between two QTL signals (e.g., edQTL and eQTL). Rather, given two traits of interest (RNA editing level and gene expression level), the colocalization analysis examines and compares the overall distribution of association *p* values for all SNPs in a large genomic window. Intuitively, a high posterior probability of colocalization is reached if the two sets of *p* value distributions track each other. We highlighted FADS1 as an example of an edQTL signal that colocalizes with its eQTL signal as well as multiple GWAS traits (Fig. 5a, b). Through differential RNA editing analysis of miRNA perturbation RNA-seq datasets, we were able to link miRNAs with potential target transcripts in an editing-specific manner. For example, we identified the edited transcript of TYK2 as a potential target of miR-138-5p. TYK2 is involved in the JAK-STAT signaling pathway and plays a critical role in the mammalian immune system [50]. When miR-138-5p was knocked down, we observed an increase in RNA editing at chr19:10462087 in TYK2 (Fig. 6c). Our results suggest that an eQTL signal of TYK2 is generated from the interaction between the edQTL at chr19:10462087 and miR-138-5p. This eQTL may give rise to multiple immune-related GWAS traits. Both the eQTL and edQTL signals colocalize with GWAS traits for systemic lupus erythematosus and neutrophil percentage in white blood cells (Fig. 6e). It should be noted that the RNA editing changes detected by RNA-seq in response to miRNA perturbation could be due to direct effects of miRNA on the degradation of edited vs unedited transcripts, or through secondary effects that are downstream of other regulatory pathways. For example, miRNA regulation of an RNA binding protein may in turn affect transcript stability in an editing-specific manner.

**Fig. 8:**
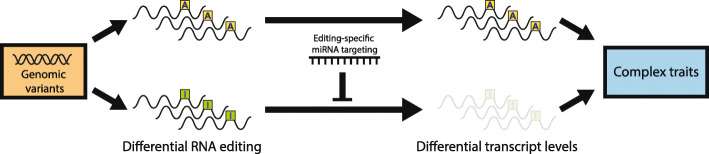
Schematic model linking edQTLs to eQTLs and complex traits. Schematic model of the regulatory mechanism in which interactions between RNA editing and miRNA-mediated transcript degradation can alter steady-state transcript levels, thus linking genomic variants with complex traits

We propose a model in which cis-regulated RNA editing events can modulate steady-state transcript levels and complex traits (Fig. 8). In the presence of a miRNA that preferentially targets the edited or unedited version of the transcript, an eQTL signal can arise from an edQTL signal through editing-specific miRNA-mediated transcript degradation. Variation in phenotypic traits could result from the varying steady-state transcript levels. This is demonstrated in the DHFR example in which miR-125a-3p links the edQTL at chr5:79923430 with the eQTL of DHFR by reducing the stability of the unedited transcripts. High DHFR expression in breast cancer has been linked to enhanced cellular proliferation and resistance to methotrexate, a chemotherapeutic agent [36]. We should note that although differential targeting of the unedited vs edited DHFR site by miR-125a-3p was known [36], our study reported several novel findings. We demonstrated that (1) the RNA editing event in DHFR is genetically controlled, (2) the edQTL signal colocalizes with the eQTL signal of DHFR, and (3) the edQTL:eQTL colocalization occurs in a miRNA concentration-dependent manner. Moreover, we show that these ingredients could come together in a single gene to create an eQTL.

To expand on the DHFR example, we carried out computational and experimental analyses to identify additional tissue-specific edQTL:edQTL colocalization events mediated by direct edQTL-miRNA interactions. By taking advantage of the comprehensive, multi-tissue edQTL dataset, we were able to (1) identify tissue-specific edQTL:eQTL colocalization events, (2) attribute some of these tissue-specific colocalization events to tissue-specific miRNA levels, and (3) experimentally confirm editing-specific miRNA-mRNA regulation using 3′-UTR luciferase reporter assays. These results demonstrate that the DHFR example generalizes to other genes and RNA editing sites.

We should note that the list of edQTL events that may generate eQTL events is expected to be substantially larger than the candidate events identified in Fig. 7. In our analysis, in order to hone into potential miRNAs computationally, we used stringent criteria to identify tissue-specific edQTL:eQTL colocalization and matched these signals with tissue-specific miRNAs identified from the GTEx data. We expect that numerous miRNAs can also generate eQTLs from edQTLs in a non-tissue-specific manner, although these candidates would be harder to identify computationally. Indeed, the DHFR example could not be identified using the computational strategy and stringent criteria outlined in Fig. 7, because all three tissues with significant edQTL signals had high or moderate colocalizing eQTL signals.

Our study focused on miRNAs as trans-acting regulators that generate eQTLs from edQTLs; however, we expect that a similar scenario could occur to RNA binding proteins that regulate RNA stability via sequence-specific protein-RNA interactions. In fact, 14% of edQTL sites (443 out of 3117) have at least one tissue in which the edQTL signal colocalizes with the eQTL signal, suggesting that a trans-acting regulator (miRNA or RNA binding protein) may alter RNA stability in an editing-specific manner. In theory, editing-specific transcript stability control by RNA binding proteins could also generate an eQTL from an existing edQTL. It has been demonstrated that ELAV1/HuR stabilizes the edited version of CTSS transcripts [51]. If ELAV1/HuR preferentially binds and stabilizes the edited or unedited version of an edQTL site, an eQTL signal should emerge. Collectively, our study reveals that RNA editing underlies a previously unappreciated mechanism for generating eQTLs in human transcriptomes, and we provide computational and experimental evidence for the role of miRNAs in creating eQTLs from edQTLs.

This work expands prior knowledge on genetically regulated RNA editing events in several major ways. Previous studies have surveyed genetically regulated RNA editing events across a limited number of cell types and tissues. In our earlier work [27], we used RNA-seq data of lymphoblastoid cell lines to find associations between genetic variation and RNA editing levels. We found evidence to support a model that cis genetic variation modulates RNA editing levels by impacting the RNA secondary structure. Furthermore, we found that some genetically regulated RNA editing events are also associated with GWAS signals, suggesting potential phenotypic consequences of RNA editing variation on complex traits and diseases. However, at the time of the study, we were unable to provide clues or suggest concrete molecular mechanisms by which genetically regulated RNA editing events affect gene products and phenotypes. Franzen and colleagues used RNA-seq data from the Stockholm-Tartu Atherosclerosis Reverse Network Engineering Task (STARNET) study to identify genetically regulated RNA editing events within individuals with coronary artery disease across seven tissues and two cell lines [47]. Similarly, the CommonMind Consortium analyzed RNA-seq data from schizophrenic individuals across two brain regions to identify genetically regulated RNA editing events that are associated with schizophrenia [48]. In this work, we analyzed population-scale RNA-seq data from 7989 samples across 49 tissues to significantly expand the catalog of genetically regulated RNA editing events in human transcriptomes. Using this comprehensive 49-tissue dataset, we were able to identify tissue-specific edQTLs and attribute the observed tissue specificity to tissue-specific expression levels of RNA editing enzymes (ADAR and ADARB1). To investigate the interplay between RNA editing variation and gene expression variation, we carried out a colocalization analysis of edQTL and eQTL signals and found evidence that cis genetic variants can causally influence RNA editing levels and gene expression levels simultaneously. Lastly, by combining computational analysis and experimental validation, we found evidence that miRNAs can generate an eQTL signal from an edQTL locus, via miRNA-mediated transcript degradation in an editing-specific manner (Figs. 7 and 8). Taken together, these results advance our conceptual understanding of the functional consequences of RNA editing and suggest that RNA editing variability can influence complex traits and diseases by altering the stability and steady-state level of critical RNA molecules.

## Conclusions

Millions of A-to-I RNA editing sites have been identified across the human transcriptome, but the functions of most RNA editing events are unknown [52]. The majority of RNA editing sites in humans are located in non-coding regions such as introns and UTRs. It is challenging to determine if a given RNA editing event is functionally relevant or if it is merely a byproduct of promiscuous editing by the ADAR enzymes. Through the lens of QTL analysis, we provide evidence that RNA editing may influence phenotypic traits by modulating steady-state transcript levels. This mechanism provides an additional layer of control in the regulation of gene expression and expands our understanding of the functional consequences of RNA editing in human cells.

## Methods

### Measuring RNA editing levels from RNA-seq datasets

For GTEx samples, RNA-seq alignments (hg19) and genotype information (GTEx_Analysis_20160115_v7_WholeGenomeSeq_635Ind_PASS_AB02_GQ20_HETX_MISS15_PLINKQC.PIR.vcf) were obtained from dbGAP (Accession phs000424.v7.p2). Alignments were downloaded using the sam-dump command from SRA-Tools [53]. Through the course of our analysis, the number of available GTEx RNA-seq samples has been continuously changing. Thus, we fixed a final set of samples (Additional file 1: Table S1) to perform our downstream analysis. We also excluded some GTEx tissues (bladder, ectocervix, endocervix, and fallopian tube) from our analysis because of low sample size. K562 samples were also excluded. Samples for which multiple RNA-seq data were generated from the same tissue and individual were pooled. When quantifying RNA editing levels, we focused our analysis on annotated RNA editing sites rather than trying to identify novel sites. A list of annotated RNA editing sites was obtained from the RNA editing ATLAS database [33] and the number of RNA-seq reads supporting the edited (G in the sense of transcription) and unedited (A in the sense of transcription) sequences were calculated for each site across each RNA-seq sample using the mpileup command from Samtools [54] (v0.1.19). We defined the editing level, Φ (frequency of inosine), as the ratio of G reads to the sum of A and G reads (*RNAeditinglevel=G/(A+G*)).

### Anatograms

Anatograms were obtained from the GTEx Portal and originated from the Expression Atlas [55], under the Creative Commons Attribution 4.0 International License.

### Preliminary filters of RNA editing sites for edQTL analysis

For any given tissue, we required the RNA editing sites to meet the following criteria: a minimum average coverage of at least two reads supporting the edited version, a minimum average total coverage of at least ten reads, and a minimum of 10% difference between the editing levels of the 90% quantile and the 10% quantile across all individuals. To remove potential artifacts, we also limited our analysis to annotated ATLAS RNA editing sites that did not overlap with annotated SNPs from the GTEx project [25], 1000 Genomes Project [56] (phase 3), or dbSNP [57] (v147).

### edQTL analysis

For each RNA editing site, we applied a linear model to SNPs within a 400-kb window centered at the editing site. We used the lm function within R to regress the editing level (Φ) against the genotype across individuals of a given tissue in order to obtain a *p* value for each SNP. To ensure accurate RNA-seq estimation of RNA editing levels, we required each sample to have a minimum coverage of 20 reads. For each SNP, we required a minor allele frequency of at least 5%. For each RNA editing site, the edQTL SNP was defined as the closest SNP with the most significant association. The number of tested sites and the number of tested site-SNP pairs per tissue are available (Additional file 12: Table S11). We used a *p* value of 1e−5 as the cutoff to call edQTL events. Using a 10% false discovery rate (FDR) threshold with a permutation procedure [58, 59] yields comparable *p* value cutoffs to 1e−5 (Additional file 13: Table S12). We defined the edQTL effect size as the slope determined from the linear model such that the *y* values are the individual editing levels (Φ) and the *x* values are the genotypes (0, 1, and 2 for Ref:Ref, Ref:Alt, and Alt:Alt, respectively).

To further investigate tissue-dependent edQTL signals in muscle vs non-muscle tissues, we fitted the data to the following multivariate model:

$$ {\varPhi}_{ij}={\mu}_i+\alpha\ {\mathrm{Genotype}}_j+\beta\ {\mathrm{Tissue}}_j+\gamma\ {\mathrm{Genotype}}_j\ast {\mathrm{Tissue}}_j+{\varepsilon}_{ij} $$

where *Φ*_*ij*_ is the RNA editing level of site *i* of sample *j*; *μ*_*i*_ is the baseline RNA editing level of site *i*; Genotype_*j*_ and Tissue_*j*_ are the genotype and tissue type of sample *j*; and *α*, *β*, and *γ* are the regression coefficients that represent the effects of genotype, tissue type, and their interaction term on RNA editing levels. Tissue_*j*_ is a binary categorical variable that represents muscle (i.e., skeletal muscle) and non-muscle tissues. To assess if the genotype effect on a given RNA editing site is tissue-dependent, we performed a likelihood ratio test by comparing the fit for the model with versus without the Genotype_*j*_ ∗ Tissue_*j*_ interaction term.

### ASED analysis

Allele-specific alignments were obtained by aligning RNA-seq reads using STAR [60] (v2.4.2a) to the hg19 genome with all heterozygous SNPs N-masked, supplied with Ensembl gene annotations (release 75) using the following alignment parameters: --alignEndsType EndToEnd --outSAMattributes NH HI NM MD --outSAMtype BAM Unsorted --outSJfilterOverhangMin 8 8 8 8 8 --outFilterType BySJout --outFilterMultimapNmax 20 --outFilterMultimapScoreRange 0 --outFilterMismatchNmax 6 --outFilterIntronMotifs RemoveNoncanonicalUnannotated --alignIntronMax 300000. In-house Python scripts [27] were used to split alignments overlapping heterozygous SNPs to the two alleles. Allele-specific read counts and Φ values were calculated from the split alignments. For each sample, we required both alleles to have non-zero coverage of RNA-seq reads and a minimum editing level of 1%. A minimum of three individuals heterozygous at the SNP location were required for subsequent analyses. We used a paired replicate statistical framework for reliable detection of allele-specific RNA editing signals in population-scale RNA-seq datasets. We treated the two alleles as matched pairs and multiple individuals sharing a given heterozygous SNP as replicates. We modeled and tested for the paired difference between the two alleles [61]. The Benjamini–Hochberg procedure was used to control the FDR at 10%.

### RNA secondary structure prediction

RNA secondary structure prediction was performed using RNAfold from the Vienna RNA Package [62] under its default parameters with the addition of the parameter --noClosingGU, which restricts GU pairs at the end of helices. Inverted Alu repeats (IRAlu) were obtained by first identifying RNA editing sites within Alu repeats and then searching for the closest neighboring Alu with the correct orientation. Alu repeats without a clear inverted partner were excluded from this analysis.

### Gene expression analysis

Gene expression values (TPM) were obtained from the GTEx Portal [25]. We used the following preprocessed GTEx datasets: “GTEx_Analysis_2016-01-15_v7_RNASeQCv1.1.8_gene_tpm.gct” was used for gene level expression (TPM) and “GTEx_Analysis_v7_eQTL_expression_matrices.tar.gz” (normalized expression) was used for eQTL plots. eQTL effect sizes (slope of the linear regression) were obtained from “GTEx_Analysis_v7_eQTL_all_associations.tar.gz”. “phe000024.v1.GTEx_ASE_SNPs.expression-matrixfmt-ase.c1.GRU.tar” was used for the allele specific expression analysis.

### edQTL non-linearity analysis

The full edQTL data were fit to a quadratic model and a linear model. The homozygous samples (Ref:Ref and Alt:Alt) were fit to a linear model. Non-linearity shifts were determined by measuring the difference between the quadratic fit (whole data) at the heterozygous genotype with the linear fit (homozygous data) at the heterozygous genotype. *p* values were obtained using a likelihood ratio test to compare the quadratic model (whole data) with the linear model (whole data).

### GWAS signals

We obtained GWAS signals from the NHGRI-EBI GWAS Catalog [63] (accessed 2019-05-03). The liftover tool from the UCSC Genome Browser [64] was used to convert hg38 genome coordinates of the GWAS Catalog to hg19 genome coordinates. VCFtools [65] was used to calculate linkage disequilibrium (LD) correlations between edQTL/ASED SNPs and GWAS SNPs. We required edQTL/ASED SNPs to be in high LD (R^2^ > 0.8) with GWAS SNPs. Genotypes from the GTEx project were used in the LD calculation. LD plots were generated with Haploview [66].

### Colocalization analysis

We used coloc [30] for 2-trait colocalization analysis and moloc [31] for 3-trait colocalization analysis. GWAS summary statistics were obtained from the NHGRI-EBI GWAS catalog [63]. edQTL summary statistics were generated with a linear model described above. eQTL summary statistics were obtained from the GTEx Portal (“GTEx_Analysis_v7_eQTL_all_associations.tar.gz”).

### miRNA expression analysis

Since direct miRNA quantifications were not available for GTEx samples, we used the number of GTEx poly-A selected RNA-seq reads that align to the cleavage product of the primary miRNA transcript normalized to the total number of aligned reads in a sample as a proxy for miRNA measurements of the mature miRNA. For a given miRNA, these values were normalized across samples such that the largest measurement was set to 1.

### Simulations of miRNA effects on edQTL and eQTL signals

For simulations in which there is no miRNA degradation effect, we set a linear relationship between the RNA editing levels across the three genotypes to simulate an edQTL signal and we set constant values for the steady-state transcript levels across the three genotypes. When we simulated the effect of miRNA-mediated transcript degradation, we chose a fixed degradation rate (either 20%, 40%, or 80%). These values were chosen to demonstrate the effect of miRNA-mediated transcript degradation on edQTL and eQTL signals across a range of degradation rates. Based on the degradation rate, either the edited transcripts or unedited transcripts were proportionally degraded. Then, the editing levels and steady-state transcript levels were computed for each genotype.

### miRNA perturbation analysis

RNA-seq reads (Additional file 9: Table S8) were aligned using STAR [60] (v2.4.2a) on to hg19 with Ensembl gene annotations (release 75). *p* values were obtained using a generalized linear mixed model [67]. FDRs were calculated using the Benjamini-Hochberg procedure. We required a change in editing level of at least 5% (|Experiment - Control|) and an FDR of ≤ 5%.

### Identification of tissue-specific edQTL:eQTL colocalization events mediated by direct edQTL-miRNA interactions

Starting from the 3117 edQTL sites, we performed edQTL:eQTL colocalization analyses tissue-by-tissue, to identify edQTL sites that meet the following criteria: (1) have at least one tissue with colocalizing edQTL and eQTL signals (PP4 > 0.75), (2) have at least one tissue with non-colocalizing edQTL and eQTL signals (PP1 > 0.75), (3) be in the 3′-UTR, and (4) have an editing-specific miRNA which is differentially expressed (fold change > 2) between the colocalizing and non-colocalizing tissues. We used TargetScan [45] to identify miRNAs that may specifically target the edited or unedited version of the mRNA, and used the GTEx RNA-seq data to quantify miRNA expression levels across human tissues.

### Cell culture and cell transfection

HEK293T cells (ATCC) were maintained in Dulbecco’s modified Eagle’s medium (DMEM) supplemented with 10% fetal bovine serum (Invitrogen). Plasmids were transiently transfected into HEK293T cells using the calcium phosphate method [68].

### Plasmid construction

To generate the dual-luciferase 3′-UTR reporter constructs for target validation, PCR fragments of unedited versions of 3′-UTRs for selected genes were amplified from SW480 genomic DNA using primers listed in Additional file 14: Table S13. PCR fragments of edited versions of 3′-UTRs with A to G mutations at selected sites were obtained by a two-step PCR method using primers listed in Additional file 14: Table S13. The PCR fragments were then inserted into pRF-con [69] (kindly provided by Dr. Ligang Wu, Shanghai Institute of Biochemistry and Cell Biology, Chinese Academy of Sciences) between *Eco*RI and *Nhe*I downstream of the Firefly luciferase ORF using Seamless Cloning Kit (D7010M, Beyotime). The regions of 3′-UTRs cloned into pRF-con and the tested RNA editing sites are listed in Additional file 15: Table S14.

To generate the expression construct of hsa-miR-4701, PCR fragment containing pri-miR-4701 was amplified from SW480 genomic DNA using primers listed in Additional file 14: Table S13. The PCR fragment was then inserted into pSIF-NEO-IRES-GFP [70] (kindly provided by Dr. Mofang Liu, Shanghai Institute of Biochemistry and Cell Biology, Chinese Academy of Sciences) between *Bam*HI and *Bgl*II using Seamless Cloning Kit. Expression constructs of hsa-miR-17, hsa-miR-125a, hsa-miR-26b, and hsa-miR-214 were kindly provided by Dr. Mofang Liu. Sequences of pri-hsa-miR-17, pri-hsa-miR-125a, pri-hsa-miR-26b, pri-hsa-miR-214, and pri-has-miR-4701 inserted in the vectors are listed in Additional file 15: Table S14.

### Dual-luciferase reporter assay

The dual-luciferase 3′-UTR reporter constructs (175 ng for DHFR, 35 ng for RPL13, 30 ng for GSR, 15 ng for MDM4 and ARPIN per well) and miRNA expression constructs or empty pSIF-NEO-IRES-GFP (each, 450 ng per well) were co-transfected into HEK293T cells in 6-well plates. Cells were scraped 32 h after transfection, and luciferase activities were measured with the Dual-Luciferase Reporter Assay System (Promega) using the GloMax™ 20/20 Luminometer (Promega) according to the manufacturer’s instructions. The Firefly luciferase activities were calculated as Firefly/Renilla luciferase values and then normalized to the empty pSIF-NEO-IRES-GFP control for each 3′-UTR reporter construct tested. Statistical analyses were performed using Student’s *t* test (*p < 0.05, **p < 0.01). Biological replicates were *n* = 3 and data were presented as the mean ± SD.

### RNA extraction and RT-qPCR

Total RNAs of HEK293T cells co-transfected with a dual-luciferase 3′-UTR reporter construct and a miRNA expression construct or empty pSIF-NEO-IRES-GFP were isolated using TRIzol™ Reagent (15596026, Invitrogen) according to manufacturer’s instructions. Total RNAs were treated with RQ1 RNase-free DNase (M6101, Promega) and then reverse-transcribed into the first-strand cDNAs using Random primer and Superscript III reverse-transcriptase (18080093, Invitrogen). The cDNAs were further analyzed by real-time PCR using iTaq™ Universal SYBR Green® Supermix (1725121, Bio-Rad) on a LightCycler® 96 Instrument (05815916001, Roche). Primers used in RT-qPCR analyses are listed in Additional file 14: Table S13. The level of the Firefly mRNA was calculated using the comparative C_T_ method relative to that of the Renilla mRNA and further normalized to the empty pSIF-NEO-IRES-GFP control for each 3′-UTR construct tested. Statistical analyses were performed using Student’s *t* test (*p < 0.05, **p < 0.01). Biological replicates were *n* = 3 and data were presented as the mean ± SD.

## Supplementary Information

**Additional file 1: Table S1.** List of analyzed datasets.

**Additional file 2: Figure S1.** Distribution of RNA editing levels (Φ) across 49 analyzed tissues. **Figure S2.** Scatter plot of the number of edQTL sites normalized by the number of tested sites vs sample size across 49 tissues. **Figure S3.** Stacked bar plots for the number and proportion of shared and tissue-specific edQTL sites and ASED sites across all tissues. **Figure S4.** Impact of edQTL SNP on RNA secondary structure and RNA editing. **Figure S5.** Tissue-dependent genotype effect sizes in muscle vs non-muscle tissues. **Figure S6.** Allele-specific expression analysis of DHFR with respect to rs1650720. **Figure S7.** Comparison of miRNA quantification between qPCR and the RNA-seq based pri-miRNA proxy method. **Figure S8.** Simulations of edQTL and eQTL signals with miRNA targeting unedited transcripts. **Figure S9.** Simulations of edQTL and eQTL signals with miRNA targeting edited transcripts. **Figure S10.** Examples of nonsynonymous edQTL sites. **Figure S11.** GWAS traits associated with edQTL and eQTL signals of FADS1. **Figure S12.** Luciferase protein expression data for validating editing-specific targeting of miRNAs to unedited or edited 3′-UTR sequences.

**Additional file 3: Table S2.** List of edQTL sites across the 49 tissues.

**Additional file 4: Table S3.** List of ASED sites across the 49 tissues.

**Additional file 5: Table S4.** List of edQTL sites with effect sizes correlated (R^2^ ≥ 0.5) to median ADARB1 levels across the 49 tissues.

**Additional file 6: Table S5.** List of nonsynonymous edQTL sites.

**Additional file 7: Table S6.** List of edQTL sites associated with GWAS traits.

**Additional file 8: Table S7.** Summary of colocalization analysis of edQTL and eQTL signals.

**Additional file 9: Table S8.** List of miRNA perturbation datasets.

**Additional file 10: Table S9.** List of differential RNA editing sites upon miRNA perturbation.

**Additional file 11: Table S10.** List of miRNA-mediated tissue-specific edQTL:eQTL colocalization events.

**Additional file 12: Table S11.** Number of tested sites and tested site-SNP pairs per tissue.

**Additional file 13: Table S12.** List of edQTL *p*-value cutoffs for FDR of 10% across the 49 tissues.

**Additional file 14: Table S13.** Primer sequences used for 3′-UTR luciferase reporter assays.

**Additional file 15: Table S14.** Description of plasmids used for 3′-UTR luciferase reporter assays.

**Additional file 16.** Review history.

## Data Availability

GTEx RNA-seq data and genotype information can be obtained from dbGAP (Accession phs000424.v7.p2). RNA editing site annotations are available at the RNA editing ATLAS (http://srv00.recas.ba.infn.it/atlas/index.html). GTEx gene expression and eQTL data can be obtained from the GTEx Portal (http://www.gtexportal.org/). miRNA perturbation RNA-seq data are listed in Additional file 9: Table S8. The GTEx edQTL and ASED data generated in this study can be explored at https://xingshiny.research.chop.edu/edqtl/. Summary statistics data for the edQTL and ASED analyses can be found at 10.5281/zenodo.4471713 [71].
