# Supplementary material for: Genetic variation and microRNA targeting of A-to-I RNA editing fine tune human tissue transcriptomes
Source: Genome Biol. 2021 Mar 9;22:77. doi: 10.1186/s13059-021-02287-1 (PMC7942016; doi:10.1186/s13059-021-02287-1)
Supplement: Supplementary file 2 — Additional file 2: Figure S1. Distribution of RNA editing levels (Φ) across 49 analyzed tissues. Figure S2. Scatter plot of the number of edQTL sites normalized by the number of tested sites vs sample size across 49 tissues. Figure S3. Stacked bar plots for the number and proportion of shared and tissue-specific edQTL sites and ASED sites across all tissues. Figure S4. Impact of edQTL SNP on RNA secondary structure and RNA editing. Figure S5. Tissue-dependent genotype effect sizes in muscle vs non-muscle tissues. Figure S6. Allele-specific expression analysis of DHFR with respect to rs1650720. Figure S7. Comparison of miRNA quantification between qPCR and the RNA-seq based pri-miRNA proxy method. Figure S8. Simulations of edQTL and eQTL signals with miRNA targeting unedited transcripts. Figure S9. Simulations of edQTL and eQTL signals with miRNA targeting edited transcripts. Figure S10. Examples of nonsynonymous edQTL sites. Figure S11. GWAS traits associated with edQTL and eQTL signals of FADS1. Figure S12. Luciferase protein expression data for validating editing-specific targeting of miRNAs to unedited or edited 3′-UTR sequences. [file 13059_2021_2287_MOESM2_ESM.pdf]

Additional File 2

Supplementary Figures S1-S12

Genetic variation and microRNA targeting of A-to-I RNA editing fine tune human tissue transcriptomes

Eddie Park, Yan Jiang, Lili Hao, Jingyi Hui, Yi Xing

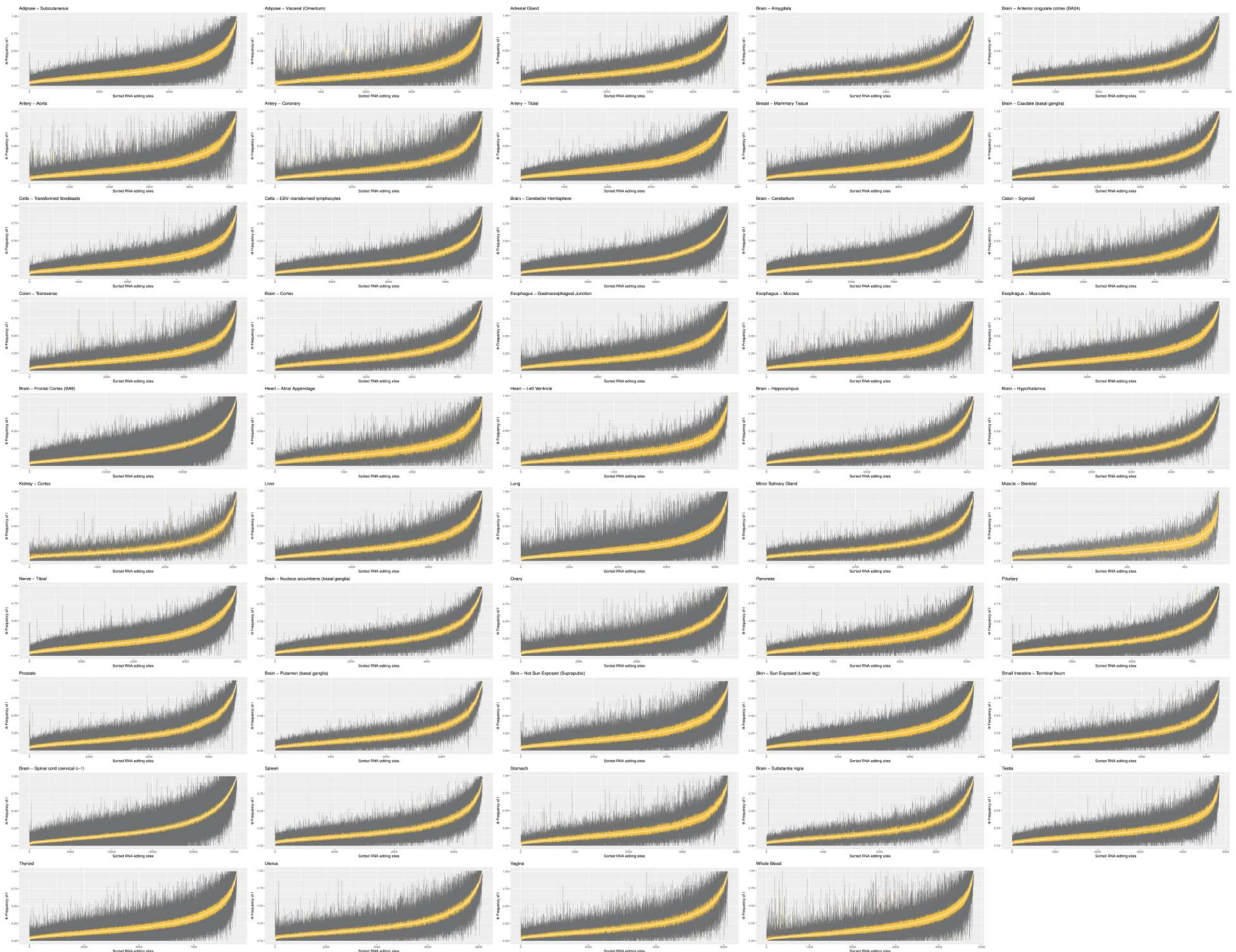

Figure S1. Distribution of RNA editing levels (Φ) across 49 analyzed tissues. Box plots show RNA editing levels of analyzed sites across all available individuals, with one box plot per site. Sites are sorted by the median Φ value on the x-axis. The interquartile ranges for each box plot are represented in orange and the medians are in white. Dark gray lines represent the whiskers of the box plots. Outliers are excluded for clarity.

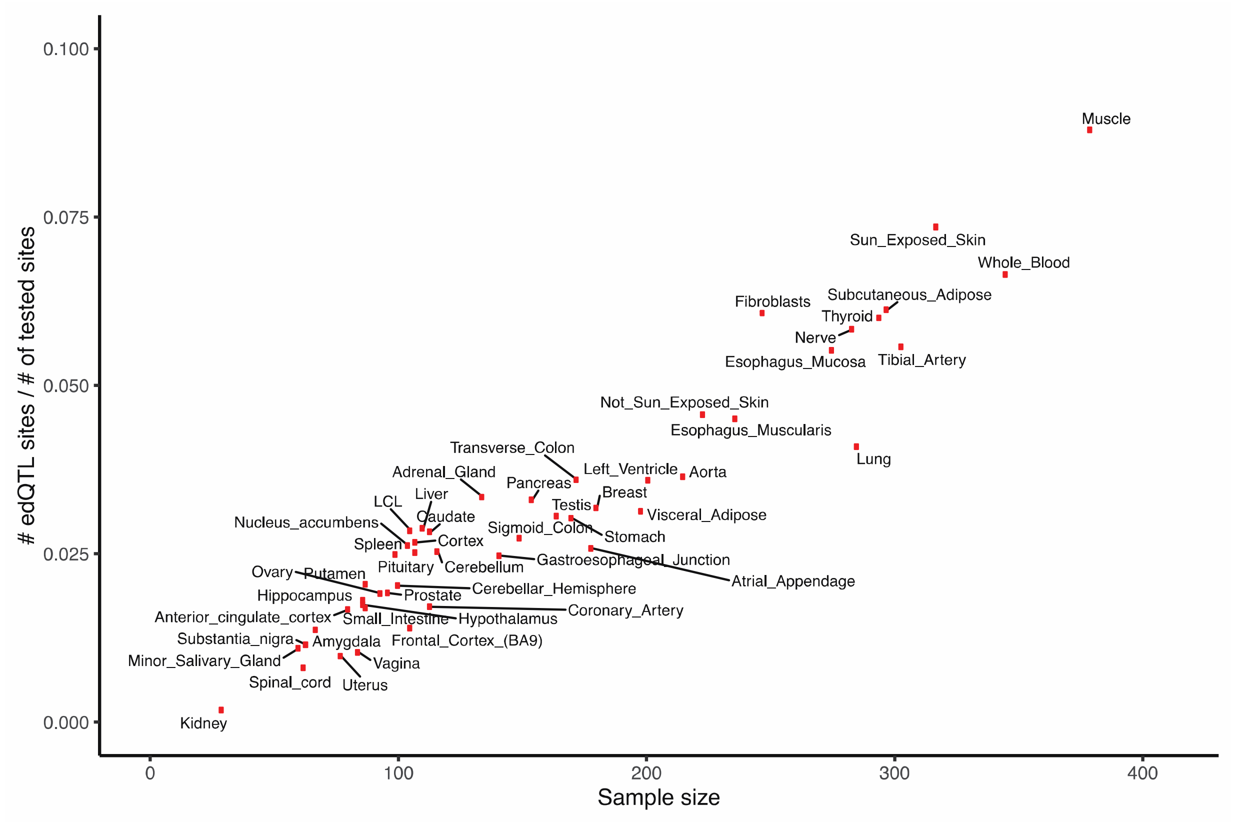

Figure S2. Scatter plot of the number of edQTL sites normalized by the number of tested sites (y-axis) vs sample size (x-axis) across 49 tissues.

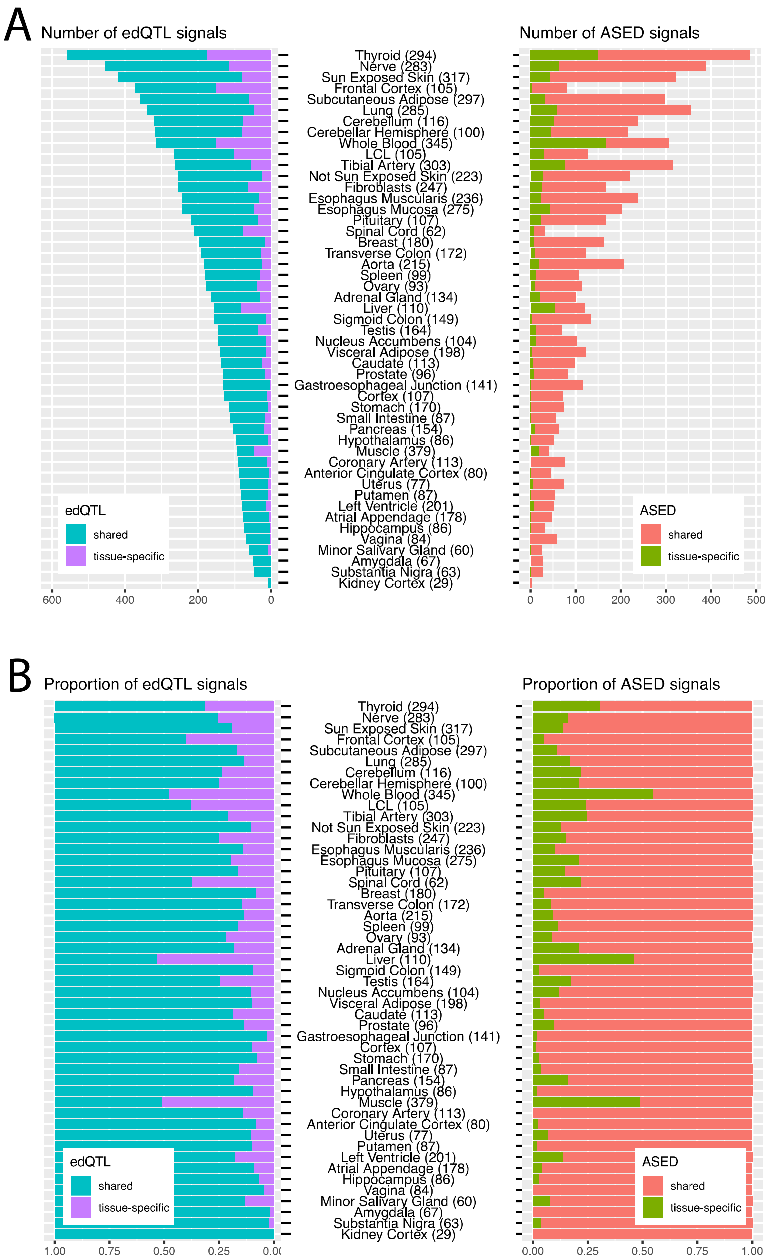

Figure S3. Stacked bar plots for the number **(A)** and proportion **(B)** of shared and tissue-specific edQTL sites (left) and ASED sites (right) across all tissues. Tissues are sorted by the number of edQTL sites.

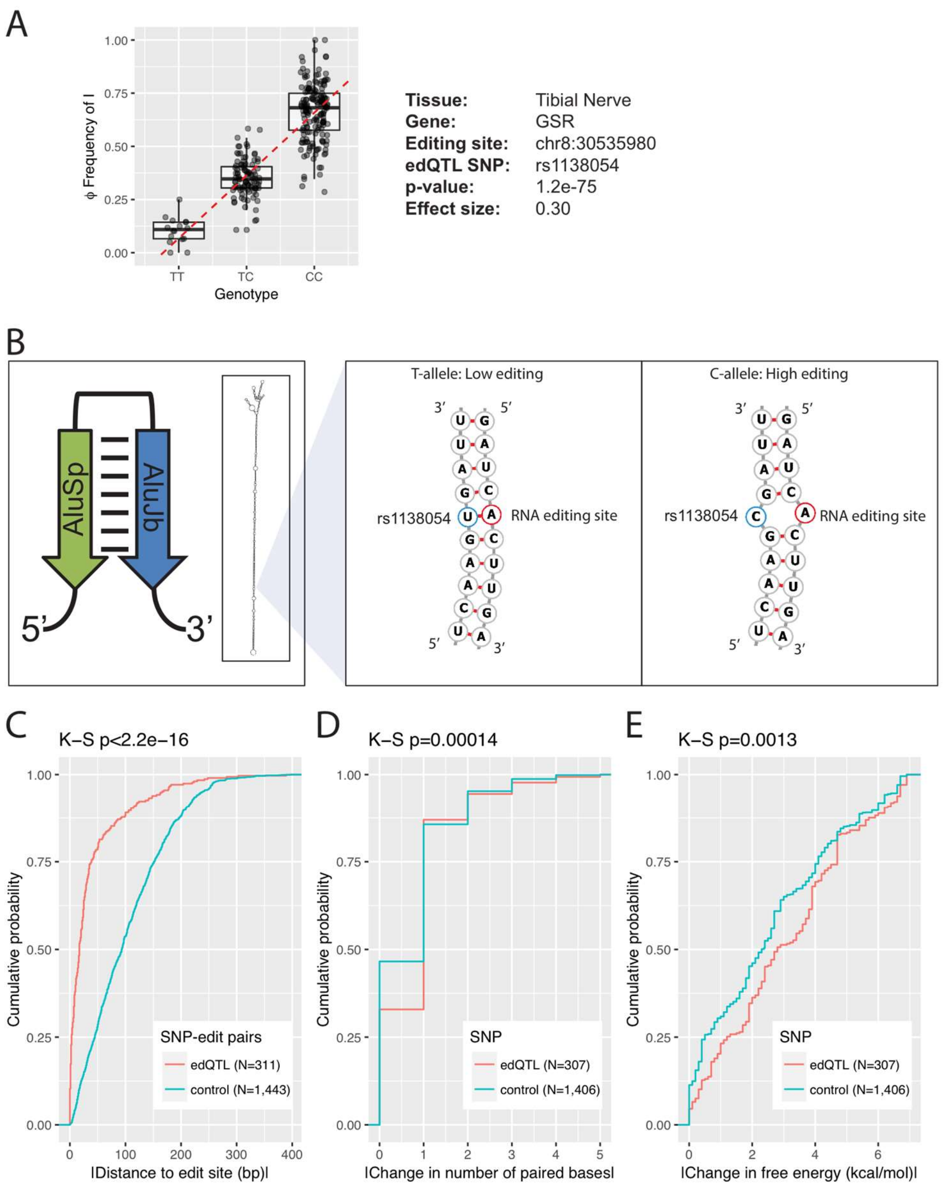

Figure S4. Impact of edQTL SNP on RNA secondary structure and RNA editing. **(A)** Box plot showing the significant association of rs1138054 with the editing level (Φ) at chr8:30535980 in GSR within the tibial nerve. Each dot represents data from a particular individual. The dashed red line represents a linear fit of the data. **(B)** Cartoon representation of IRAlu hairpin and computationally predicted RNA secondary structure (left). Detailed base-pairing structures of the edQTL SNP with the RNA editing site are shown (right). **(C)** Cumulative distribution plot comparing the absolute value of the distance between SNP-RNA editing site pairs for significant edQTL SNPs and control SNPs within the computationally predicted RNA secondary structure of the IRAlu hairpin. **(D)** Cumulative distribution plot comparing the absolute value of the change in the number of paired bases for significant edQTL SNPs and control SNPs. **(E)** Cumulative distribution plot comparing the absolute value of the change in free energy of the predicted RNA secondary structure for significant edQTL SNPs and control SNPs. The Kolmogorov-Smirnov test was used for the cumulative distribution plots.

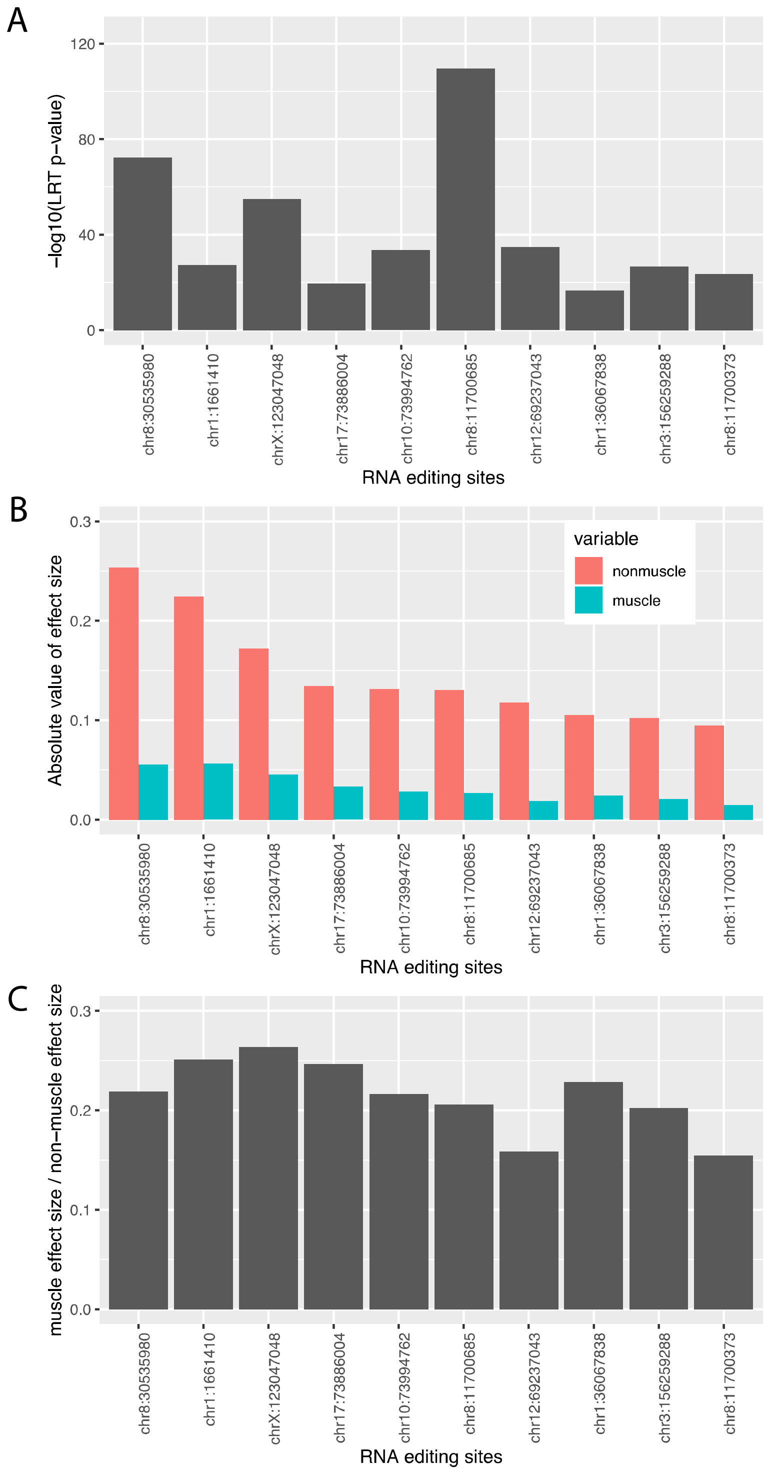

Figure S5. Tissue-dependent genotype effect sizes in muscle vs non-muscle tissues. **(A)** Bar plot of the -log10(likelihood ratio test p-value) for the significance of the genotype x tissue interaction term on RNA editing levels. **(B)** Bar plot of the absolute value of genotype effect sizes in muscle and non-muscle tissues. **(C)** Bar plot of the ratio between genotype effect sizes in muscle and non-muscle tissues.

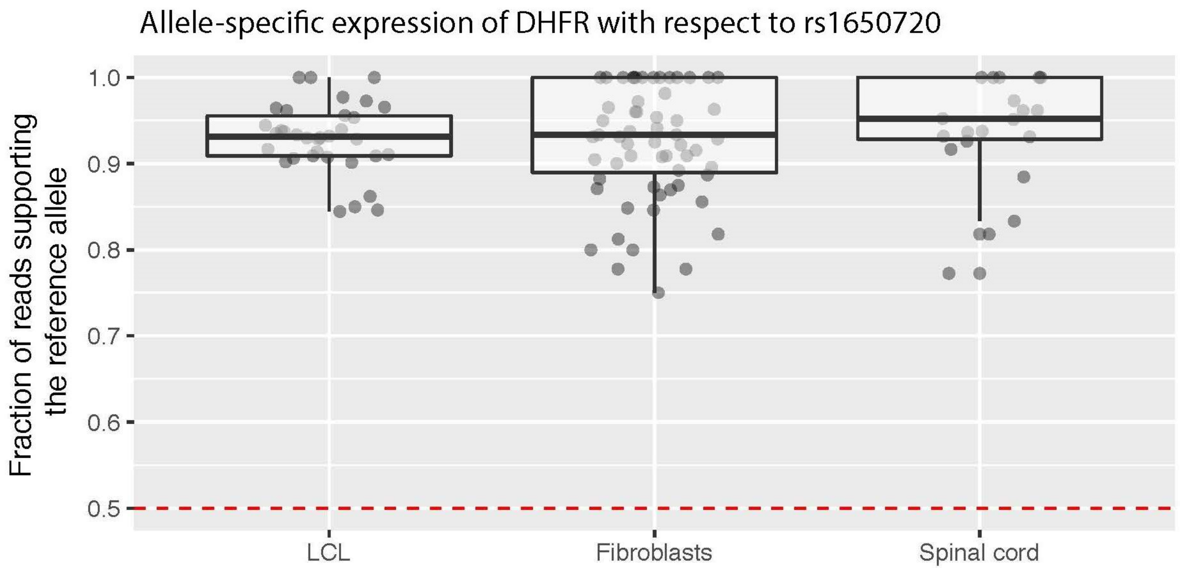

Figure S6. Allele-specific expression analysis of DHFR with respect to rs1650720. The fraction of reads supporting the reference allele is plotted for each tissue. The red dotted line at 0.5 indicates the level at which there is an equal number of reads for each allele.

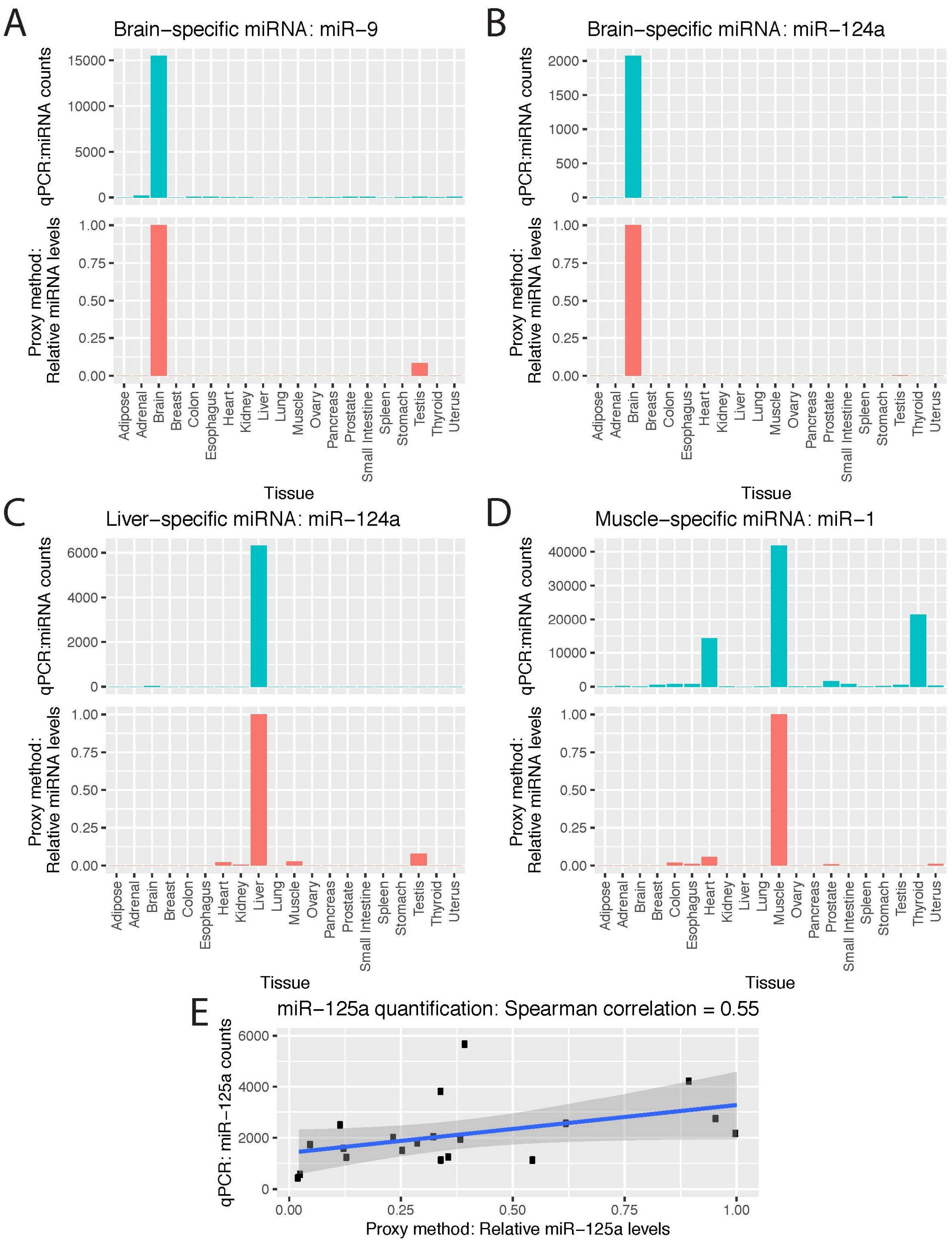

Figure S7. Comparison of miRNA quantification between qPCR and the RNA-seq based pri-miRNA proxy method. Bar plots comparing qPCR and the proxy methods for **(A)** brain-specific miR-9, **(B)** brain-specific miR-124a, **(C)** liver-specific miR-122a, and **(D)** muscle-specific miR-1. (E) Scatter plot of miR-125a levels estimated by qPCR and the proxy method for a set of tissues shared between the two datasets. The blue line represents a linear fit of the data and the gray region represents the 95% confidence interval.

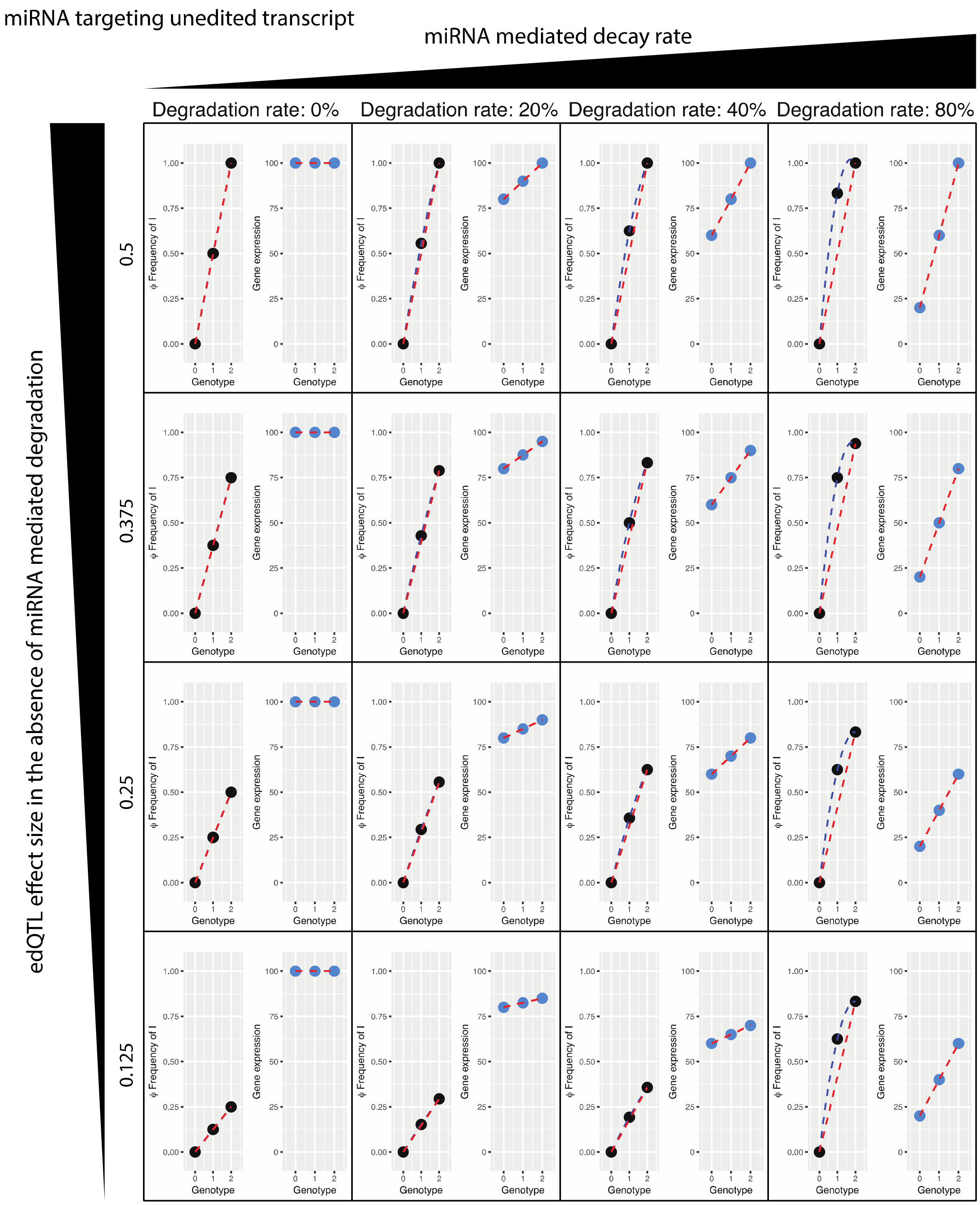

Figure S8. Simulations of edQTL and eQTL signals with miRNA targeting unedited transcripts. Simulations of edQTL and eQTL signals when a miRNA targets the unedited transcripts. Varying miRNA-mediated transcript degradation rates of 0%, 20%, 40%, and 80% were tested. Varying edQTL effect sizes of 0.5, 0.375, 0.25, and 0.125 were tested. Dashed blue lines represent quadratic fits of the data. Dashed red lines represent linear fits of the data.

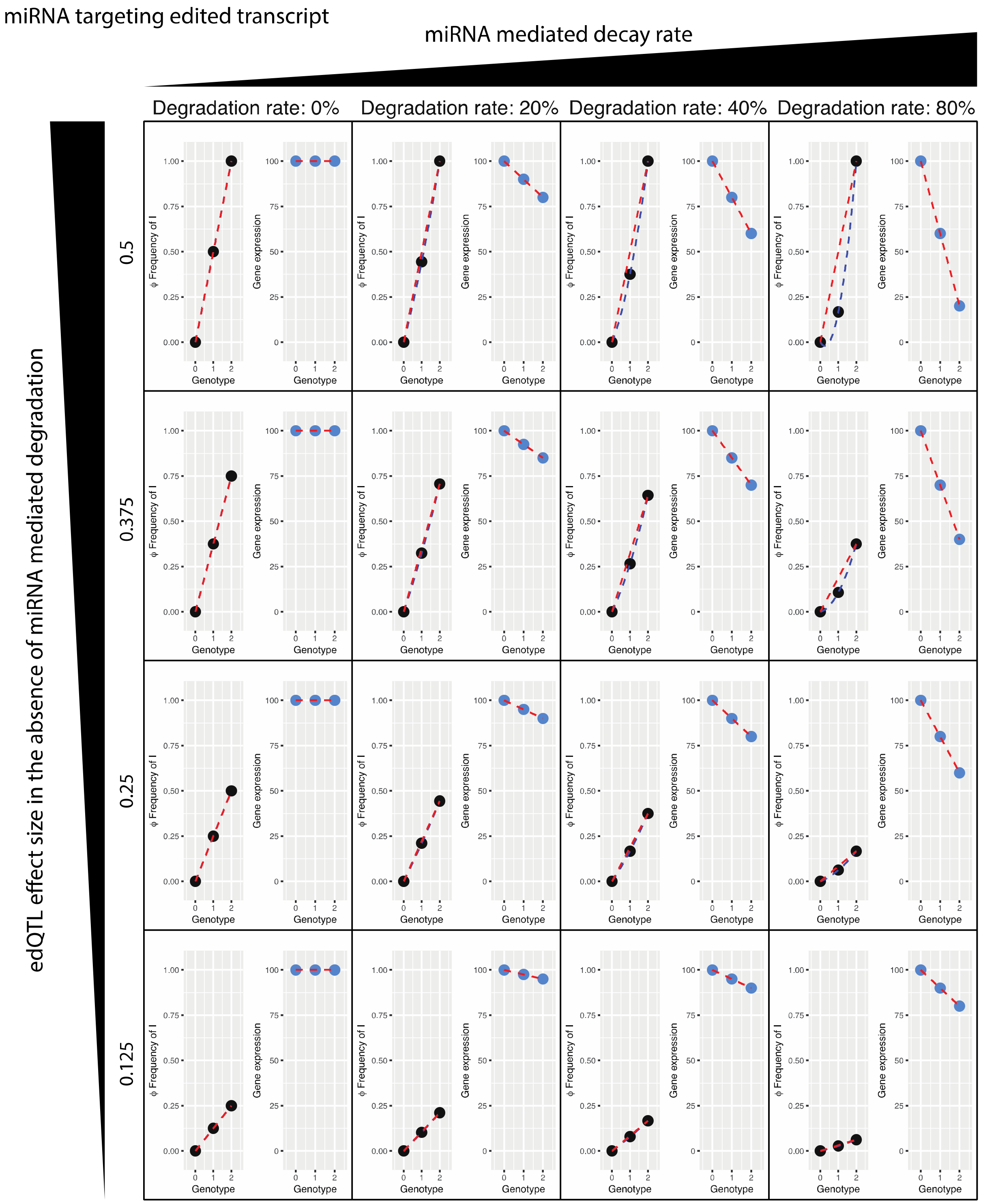

Figure S9. Simulations of edQTL and eQTL signals with miRNA targeting edited transcripts. Simulations of edQTL and eQTL signals when a miRNA targets the edited transcripts. Varying miRNA-mediated transcript degradation rates of 0%, 20%, 40%, and 80% were tested. Varying edQTL effect sizes of 0.5, 0.375, 0.25, and 0.125 were tested. Dashed blue lines represent quadratic fits of the data. Dashed red lines represent linear fits of the data.

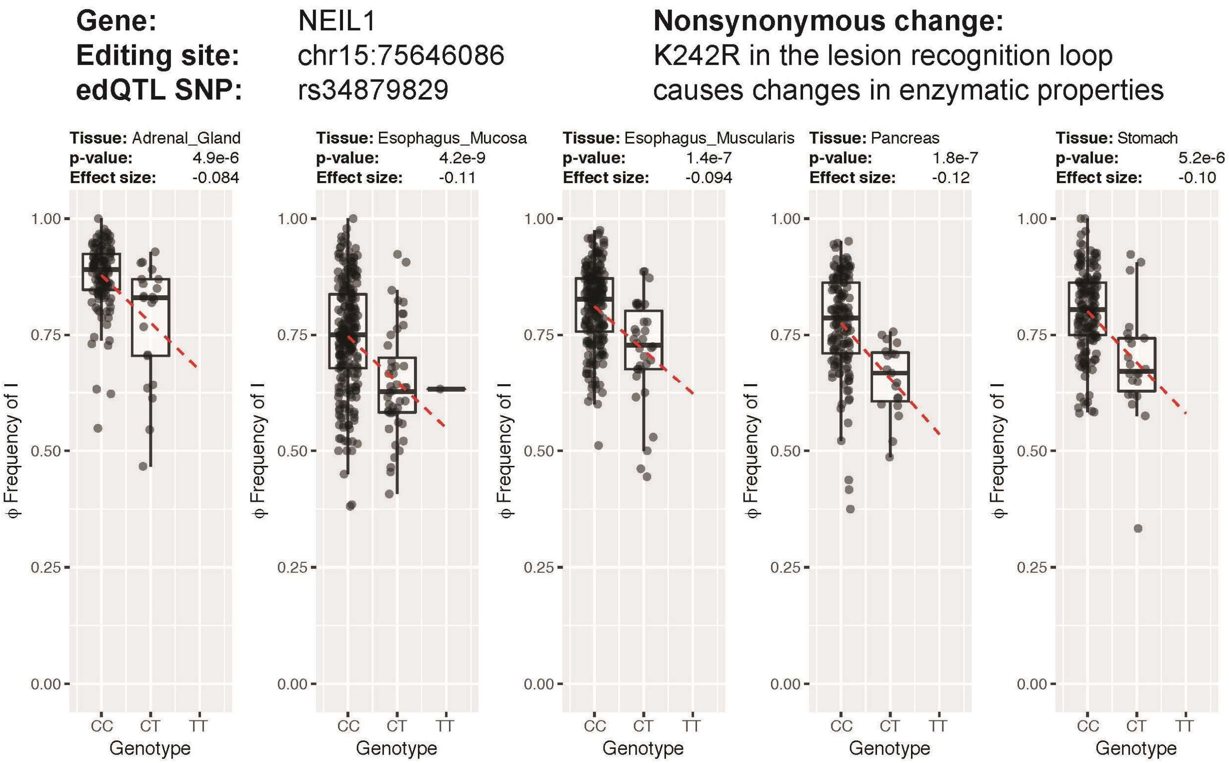

Figure S10. Examples of nonsynonymous edQTL sites. RNA editing at chr15:75646086 results in a K242R amino acid change, which results in altered enzymatic properties of the DNA repair enzyme NEIL1. Box plots show the significant association of rs34879829 with the editing level (Φ) at chr15:75646086 in NEIL1 within the adrenal gland, esophagus mucosa, esophagus muscularis, pancreas, and stomach. Each dot represents data from a particular individual. The dashed red line represents a linear fit of the data.

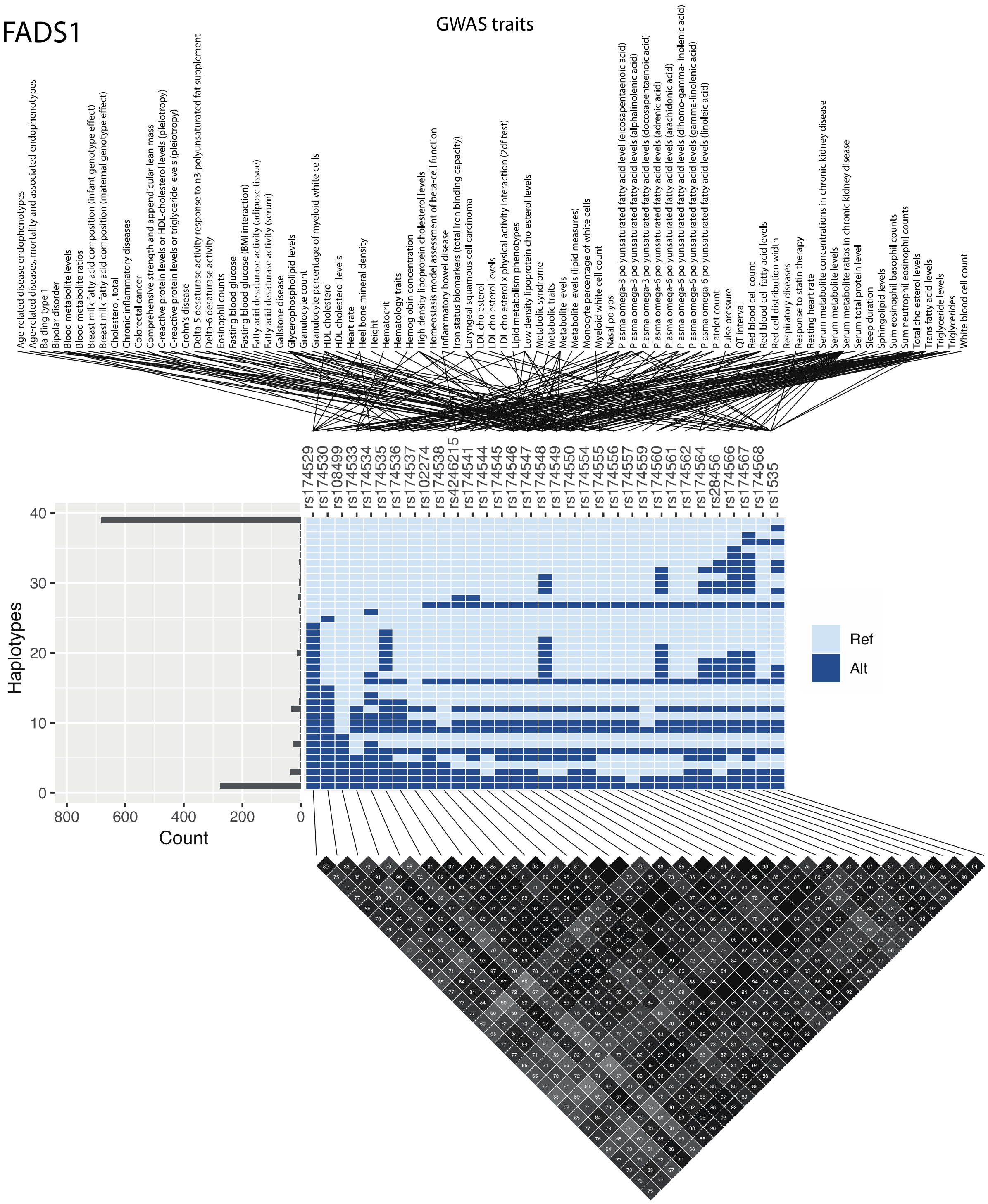

Figure S11. GWAS traits associated with edQTL and eQTL signals of FADS1. Full list of GWAS traits associated with the edQTL at chr11:61567758 and the eQTL of FADS1 (top). Histogram and heatmap illustrate haplotypes observed in GTEx samples (middle). LD plot shows linkage disequilibrium between GWAS SNPs.

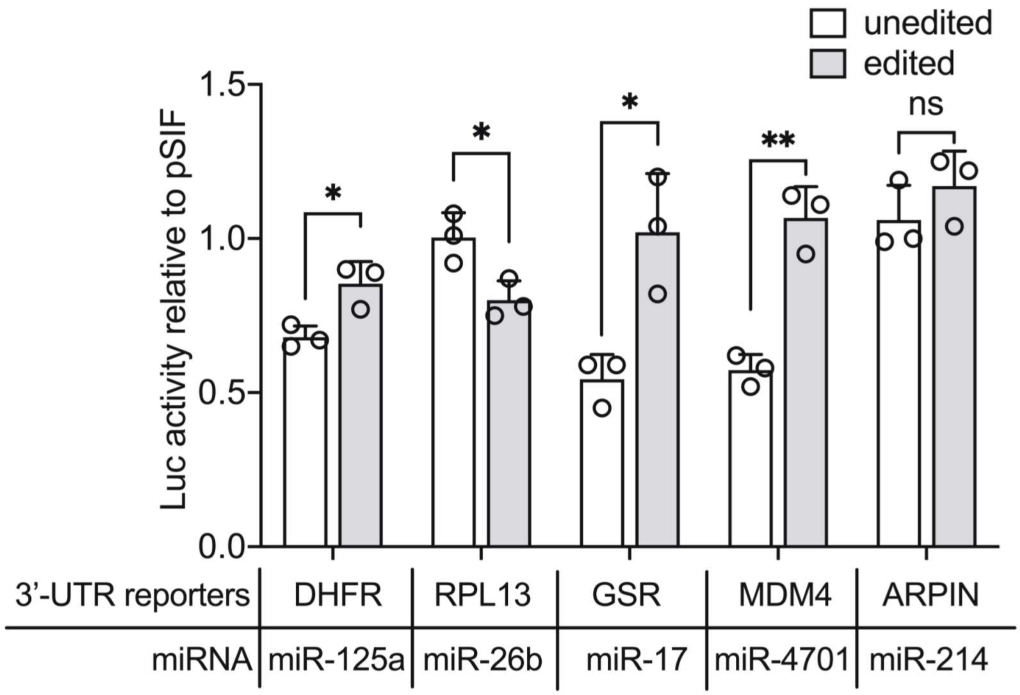

Figure S12. Luciferase protein expression data for validating editing-specific targeting of miRNAs to unedited or edited 3'-UTR sequences. Each barplot displays the mean and SD of three independent experiments. * $p<0.05$, ** $p<0.01$.
