# Supplementary material for: Genetic variation and microRNA targeting of A-to-I RNA editing fine tune human tissue transcriptomes
Source: Genome Biol. 2021 Mar 9;22:77. doi: 10.1186/s13059-021-02287-1 (PMC7942016; doi:10.1186/s13059-021-02287-1)
Supplement: Supplementary file 16 — Additional file 16. Review history. [file 13059_2021_2287_MOESM16_ESM.docx]

Review History

**First round of review**

**Reviewer 1**

**Are you able to assess all statistics in the manuscript, including the appropriateness of statistical tests used?** Yes, and I have assessed the statistics in my report.

**Comments to author:**

The authors have extended an earlier work (PMID: 28754146; GB 2017) and looked for SNPs affecting editing levels using the full GTEx dataset. The link between genetic variation and editing is indeed very interesting. However, the progress made in the present study (compared to the previous one by the same group and similar studies from other groups) is, in my opinion, incremental.

Given the above, I have not looked into the results in much detail. However, here are some points that may be helpful in preparing for submission to a different journal:

1. As noticed by the authors, observing fewer edQTLs in muscle is only expected, due to the overall lower editing there. It might be useful to check whether the difference is indeed fully explained by the lower editing level, though. One possible model to consider is that of a multiplicative effect, where editing level is the product of a tissue-dependent and site dependent editing efficiency, and the genotype contribution. Is there any dependence on ADAR levels beyond this trivial effect?

2. Same goes for "missing associations". Are they really missing, or is it just a case of an overall low editing rate in the specific site and tissue?

3. The nonlinear dependence is a nicely explained anecdote, please supplement it with allele-specific expression levels.

4. The effect of editing in microRNA targets was also discussed in the literature. The examples provided here are interesting. GTEx data studied here contain miRNA expression profiles. Do you see a correlation between the miRNA level and the editing level in this dataset too?

5. Analyze computationally predicted (putative) secondary structure systematically, to show the edQTLs (or at least many of them) do have a sizable impact on the structure. This direction may lead to novel discoveries regarding the rules controlling editing efficiency.

**Reviewer 2**

**Are you able to assess all statistics in the manuscript, including the appropriateness of statistical tests used?** Yes, and I have assessed the statistics in my report.

**Comments to author:**

In the manuscript entitled "Genetic variation and microRNA targeting of A-to-I RNA editing fine tune human tissue transcriptomes", Park et al. identified edQTL events in 49 tissues across 437 individuals. They observed that certain microRNAs are able to differentiate between the edited and unedited isoforms of their targets, and microRNAs can generate eQTL signal from an edQTL locus by microRNA-mediated transcript degradation through the regulation of RNA editing. They also found that some of edQTLs are associated with GWAS signals. These results provide an additional layer of control in the regulation of gene expression and expands our understanding of the functional consequences of RNA editing in human cells.

This work is potentially of broad interest to RNA editing, microRNA, QTL, and genetic variants community in that the authors showed a mechanism in which RNA editing variability can influence the phenotypes of complex traits and diseases by altering the stability and steady-state level of critical RNA molecules. This study is novel and interesting, especially in the broad area of genetic architecture. The methods used are solid and the data are also well presented and discussed.

Major comments:

1. In the miRNA expression analysis, the authors used RNA-seq reads that align to the primary miRNA transcript as a proxy for miRNA measurement of the mature form. It is little concern that if the primary miRNA transcript can reflect mature miRNA expression. It is better for the authors to validate by using independent dataset.

2. In line 119-130, the authors found 3,117 unique RNA editing sites that are associated with at least one edQTL SNP across 49 tissues, and showed the number of RNA editing sites in each tissue. Do these RNA editing sites show tissue-specific or shared across tissues? How many edQTL SNPs are associated with RNA editing sites in each tissue? The similar analysis is needed in ASED analysis.

Minor comments that may need to be clarified:

1. In the simulation of miRNA effects on edQTL and eQTL signal, could the authors explain more clearly why to select a fixed degradation at 20%, 40%, and 80%?

2. In line 110-111, how to define a variable RNA editing site? Based on the difference of RNA editing level?

3. In line 392 please explain clearly how to use the linear model to perform edQTL analysis.

4. In line 167, "In some tissues, RNA editing is associated with rs2707852." This sentence seems not completed.

5. In figure 2D, 3A, 3E, 4A, 5A, and 6D, please mark how many samples are in each genotype. In the Figure 4A, it seems that the sample size for AA genotype is small.

6. In line 232-233, the authors mentioned that "this suggests a possible genetic mechanism affecting predispositions to cancer", they use the GTEx tissues to quantify the edQTL. How can this linked to cancer?

**Authors Response**

**Point-by-point responses to the reviewers’ comments:**

***Reviewer #1:***

*The authors have extended an earlier work (PMID: 28754146; GB 2017) and looked for SNPs affecting editing levels using the full GTEx dataset. The link between genetic variation and editing is indeed very interesting. However, the progress made in the present study (compared to the previous one by the same group and similar studies from other groups) is, in my opinion, incremental. Given the above, I have not looked into the results in much detail. However, here are some points that may be helpful in preparing for submission to a different journal:*

We thank the reviewer for the constructive comments and suggestions on our work. The reviewer felt that our work is “incremental” over our earlier publication (PMID: 28754146; GB 2017) as well as similar studies from other groups. We respectfully disagree and believe that this work represents significance advances over existing literature on genetically regulated RNA editing events. Nonetheless, in light of the reviewer’s opinion, we have expanded the discussions in this manuscript, to highlight the novelty and significance of this new work and how it differs and advances from previous studies including our 2017 publication.

In our earlier work (PMID: 28754146; GB 2017), we used RNA-seq data from lymphoblastoid cell lines to find associations between genetic variation and RNA editing levels. We found evidence to support a model that cis genetic variation modulates RNA editing levels by impacting the RNA secondary structure. Furthermore, we found that some genetically regulated RNA editing events are also associated with GWAS signals, suggesting potential phenotypic consequences of RNA editing variation on complex traits and diseases. However, at the time of the study, we were unable to provide any clues or suggest concrete molecular mechanisms by which genetically regulated RNA editing events affect gene products and phenotypes. Additionally, given that our earlier study was conducted on a single cell line (lymphoblastoid cell line), the catalog of genetically regulated RNA editing events was rather limited, and we were unable to investigate tissue variation and specificity of these RNA editing events.

This current work differs from our previous publication and similar studies from other groups in several major ways:

- We analyzed population-scale RNA-seq data of 7,989 samples across 49 tissues, significantly expanding the catalog of genetically regulated RNA editing events in human transcriptomes.
- Using this comprehensive dataset, we were able to identify tissue-specific edQTLs. Furthermore, we observed that this tissue-specificity can be explained in part by tissue-specific expression levels of RNA editing enzymes (ADAR and ADARB1).
- To the best of our knowledge, this work is the first to perform colocalization analyses of edQTL and eQTL signals. Importantly, we found evidence that cis genetic variants can causally influence RNA editing levels and gene expression levels simultaneously, suggesting the interplay between edQTL and eQTL signals.
- By combining computational modeling with empirical evidence based on miRNA perturbation RNA-seq experiments, we suggest a molecular mechanism that miRNAs can generate an eQTL signal from an edQTL locus, via miRNA-mediated transcript degradation in an editing-specific manner. This advances our conceptual understanding of the functional consequences of RNA editing, and suggest that RNA editing variability can influence complex traits and diseases, by altering the stability and steady-state level of critical RNA molecules.

To highlight the novelty and significance of this work, we added the following paragraph to the Discussion section of the revised manuscript (Page 17-19):

**“This work expands prior knowledge on genetically regulated RNA editing events in several major ways. Previous studies have surveyed genetically regulated RNA editing events across a limited number of cell types and tissues. In our earlier work (Park 2017), we used RNA-seq data of lymphoblastoid cell lines to find associations between genetic variation and RNA editing levels. We found evidence to support a model that cis genetic variation modulates RNA editing levels by impacting the RNA secondary structure. Furthermore, we found that some genetically regulated RNA editing events are also associated with GWAS signals, suggesting potential phenotypic consequences of RNA editing variation on complex traits and diseases. However, at the time of the study, we were unable to provide clues or suggest concrete molecular mechanisms by which genetically regulated RNA editing events affect gene products and phenotypes. Franzen and colleagues used RNA-seq data from the Stockholm-Tartu Atherosclerosis Reverse Network Engineering Task (STARNET) study to identify genetically regulated RNA editing events within individuals with coronary artery disease across seven tissues and two cell lines (Franzen 2018). Similarly, the CommonMind Consortium analyzed RNA-seq data from schizophrenic individuals across two brain regions to identify genetically regulated RNA editing events that are associated with schizophrenia (Breen 2019). In this work, we analyzed population-scale RNA-seq data from 7,989 samples across 49 tissues to significantly expand the catalog of genetically regulated RNA editing events in human transcriptomes. Using this comprehensive 49-tissue dataset, we were able to identify tissue-specific edQTLs and attribute the observed tissue specificity to tissue-specific expression levels of RNA editing enzymes (ADAR and ADARB1). To investigate the interplay between RNA editing variation and gene expression variation, we carried out a colocalization analysis of edQTL and eQTL signals and found evidence that cis genetic variants can causally influence RNA editing levels and gene expression levels simultaneously. Lastly, by combining computational modeling with empirical evidence based on miRNA perturbation RNA-seq experiments, we suggest a molecular mechanism that miRNAs can generate an eQTL signal from an edQTL locus, via miRNA-mediated transcript degradation in an editing-specific manner. This advances our conceptual understanding of the functional consequences of RNA editing, and suggest that RNA editing variability can influence complex traits and diseases by altering the stability and steady-state level of critical RNA molecules.”**

*1. As noticed by the authors, observing fewer edQTLs in muscle is only expected, due to the overall lower editing there. It might be useful to check whether the difference is indeed fully explained by the lower editing level, though. One possible model to consider is that of a multiplicative effect, where editing level is the product of a tissue-dependent and site dependent editing efficiency, and the genotype contribution. Is there any dependence on ADAR levels beyond this trivial effect?*

In fact, our data suggest that the tissue-specific variation of edQTL signals in muscle vs non-muscle tissues is not only due to the low baseline RNA editing levels in muscle, but also due to the smaller genotype effect sizes in muscle. We thank the reviewer for the comment and suggestion, and we appreciate the opportunity to clarify this issue and provide additional supporting evidence in our revision.

In the original manuscript, we performed edQTL analyses one tissue at a time, and detected fewer than expected number of edQTL sites in muscle. Then, for each tissue where an RNA editing site had sufficient RNA-seq coverage, we estimated the genotype effect size on RNA editing levels in that tissue. This analysis indicates that edQTL sites in muscle tend to have smaller genotype effect sizes than in other tissues (Figure 3C). An example is an RNA editing site in the gene GSR. At this site, the baseline RNA editing levels in muscle and non-muscle tissues (nucleus accumbens, for example) are comparable (low in both tissue types), while the genotype effect size is significantly smaller in muscle compared to nucleus accumbens (Figure 3A).

To further investigate this issue, following the reviewer’s suggestion we adopted a complementary approach to investigate tissue-dependent edQTL signals in muscle vs non-muscle tissues, by using a multivariate model. Specifically, we fitted the data to the following model:

where _Φij_ is the RNA editing level of site i of sample j; μ_i_ is the baseline RNA editing level of site i; Genotype_j_ and Tissue_j_ are the genotype and tissue type of sample j; and α, β, and γ are the regression coefficients that represent the effects of genotype, tissue type, and their interaction term on RNA editing levels. Tissue_j_ is a binary categorical variable that represents muscle (i.e. skeletal muscle) and non-muscle tissues. To assess if the genotype effect on a given RNA editing site is tissue-dependent, we performed a likelihood ratio test by comparing the fit for the model with versus without the Genotype_j_ ∗ Tissue_j_ interaction term.

We applied this multivariate model and likelihood ratio test to the 10 edQTL RNA editing sites with the largest average genotype effect size across 49 tissues among the 227 sites used to generate Figure 3C (i.e. edQTL RNA editing sites with sufficient RNA-seq coverage and measurable effect sizes in all 49 tissues). We chose to focus on the top 10 edQTL sites to obtain a high signal-to-noise ratio in the estimated effect sizes, so we can reliably dissect genotype and tissue effects on RNA editing levels. For all 10 sites, the likelihood ratio test for the interaction term is highly significant (Additional file 8: Figure S5A), suggesting that the genotype effect on RNA editing levels is tissue-dependent and differs significantly between muscle vs non-muscle tissues. Based on the estimated effect sizes in muscle vs non-muscle tissues, we found that muscle typically has an 80% reduction in genotype effect size compared to non-muscle tissues (Additional file 8: Figure S5B-C). This result is consistent with the example described above for the GSR RNA editing site (Figure 3A). Taken together, these data suggest that the effects of genotype and tissue type on RNA editing levels go beyond a trivial independent model and the genotype effect size can vary significantly across tissues.

We added the following text to Page 8 and 21 of the revised manuscript to describe this new analysis, along with a new supplemental figure (Additional file 8: Figure S5):

Page 8:

**“To more formally assess if the genotype effect on a given RNA editing site is tissue-dependent (muscle vs non-muscle), we applied a multivariate model and likelihood ratio test to the 10 edQTL RNA editing sites with the largest average genotype effect size across**

**49 tissues among the 227 sites used to generate Figure 3C (i.e. edQTL RNA editing sites with sufficient RNA-seq coverage and measurable effect sizes in all 49 tissues). We chose to focus on the top 10 edQTL sites to obtain a high signal-to-noise ratio in the estimated effect sizes, so we can reliably dissect genotype and tissue effects on RNA editing levels. For all 10 sites, the likelihood ratio test for the genotype x tissue interaction term is highly significant (Additional file 8: Figure S5A), suggesting that the genotype effect on RNA editing levels is tissue-dependent and differs significantly between muscle vs non-muscle tissues. Based on the estimated effect sizes in muscle vs non-muscle tissues, we found that muscle typically has an 80% reduction in genotype effect size compared to non-muscle tissues (Additional file 8: Figure S5B-C).”**

**“To further investigate tissue-dependent edQTL signals in muscle vs non-muscle tissues, we fitted the data to the following multivariate model:**

Page 21:

Additional file 8: Figure S5. Tissue-dependent genotype effect sizes in muscle vs non-muscle tissues.

(A) Bar plot of the -log10(likelihood ratio test p-value) for the significance of the genotype x tissue interaction term on RNA editing levels.
(B) Bar plot of the absolute value of genotype effect sizes in muscle and non-muscle tissues.
(C) Bar plot of the ratio between genotype effect sizes in muscle and non-muscle tissues.

**where Φ_ij_ is the RNA editing level of site i of sample j; µ_i_ is the baseline RNA editing level of site i; Genotpe _j_ and Tissue _j_ are the genotype and tissue type of sample j; and α, β, and y are the regression coefficients that represent the effects of genotype, tissue type, and their interaction term on RNA editing levels. Tissue] is a binary categorical variable that represents muscle (i.e. skeletal muscle) and non-muscle tissues. To assess if the genotype effect on a given RNA editing site is tissue-dependent, we performed a likelihood ratio test by comparing the fit for the model with versus without the Genotype_]_ * Tissue_]_ interaction term.”**

2. *Same goes for "missing associations". Are they really missing, or is it just a case of an overall low editing rate in the specific site and tissue?*

Our original manuscript did not contain the phrase “missing associations”. However, the phrase “this association is missing” was used to refer to the example in Figure 3E, so we assume the reviewer was referring to this example. For this RNA editing site (in RABGEF1), a significant genotype effect on RNA editing levels was only observed in tissues with sufficient ADARB1 levels (Figure 3F). The tibial artery had a high level of ADARB1 while whole blood had a low level. Consistent with the ADARB1 levels, the tibial artery had a strong edQTL signal while the edQTL signal in whole blood was non-existent (Figure 3E). In response to the reviewer’s question, we similarly applied the multivariate model and likelihood ratio test (described above) to this site, with tissues grouped into those with high (top 4 tissues as highlighted in Figure 3F) vs low (the rest 45 tissues) ADARB1 levels. The likelihood ratio test for the genotype x tissue interaction term is highly significant (p-value< 2.2e-16), indicating that for this particular site, not only the baseline RNA editing levels but also the genotype effect sizes on RNA editing levels differ between tissues with high vs low ADARB1 levels.

Nonetheless, we recognize that “missing association” could be potentially confusing in the human genetics literature, therefore we modified this sentence to read as: **“RNA editing is significantly associated with rs2707852 in some tissues but not in others.”**

3. *The nonlinear dependence is a nicely explained anecdote, please supplement it with allele-specific expression levels.*

We appreciate the reviewer’s positive comment. As requested, we have supplemented this example with allele-specific expression levels of DHFR with respect to rs1650720 (Additional file 10: Figure S6). As expected, we observed allele-specific expression towards the reference allele in all three tissues. We added the following text to Page 11 of the revised manuscript to describe the allele-specific expression data:

**“Furthermore, allele-specific expression (ASE) analysis confirms higher expression of the reference allele in all three tissues (Additional file 10: Figure S6).”**

Additional file 10: Figure S6. Allele-specific expression analysis of DHFR with respect to rs1650720. The fraction of reads supporting the reference allele is plotted for each tissue. The red dotted line at 0.5 indicates the level at which there is an equal number of reads for each allele.

4. *The effect of editing in microRNA targets was also discussed in the literature. The examples provided here are interesting. GTEx data studied here contain miRNA expression profiles. Do you see a correlation between the miRNA level and the editing level in this dataset too?*

We appreciate the reviewer’s positive comment that “examples provided here are interesting”. It is true that the potential of RNA editing at mRNA 3’-UTR to modulate miRNA targeting has been discussed in the literature. However, to our knowledge, except for anecdotal examples in specific genes these were primarily speculations based on overlapping RNA editing sites and putative miRNA target sites. In our work, we used data from miRNA perturbation RNA-seq experiments to show a change in the steady state RNA editing levels of many RNA editing sites, providing empirical evidence that the perturbed miRNA could preferentially target the edited or unedited version of the transcript.

It is our understanding that the GTEx consortium did not generate miRNA expression profiles. The data generated by the GTEx consortium is from long RNA-seq using poly-A selected RNAs. A small RNA library preparation is required to enrich and profile mature processed miRNAs. While it is true that miRNA genes (most of these are annotations for the pre-miRNA hairpins) have been quantified and reported in the compiled gene expression tables from GTEx, the majority of these values are zero or near-zero because the RNA-seq method used by GTEx does not capture mature miRNAs. Thus, these values do not reflect the levels of mature miRNAs in the GTEx tissues. We should also note that the lack of small RNA/miRNA data in GTEx was also corroborated (albeit informally) in a recent Twitter Q&A by Dr. Tuuli Lappalainen, a core member and leader of GTEx (see <https://twitter.com/tuuliel/status/1166738878029602817).>

5. *Analyze computationally predicted (putative) secondary structure systematically, to show the edQTLs (or at least many of them) do have a sizable impact on the structure. This direction may lead to novel discoveries regarding the rules controlling editing efficiency.*

We thank the reviewer for this suggestion. Understanding the rules controlling editing efficiency would be interesting and important. We would like to note that we had performed analyses into this topic in our earlier GB 2017 publication. Specifically, in our previous work, we analyzed computationally predicted RNA secondary structures and found that edQTL SNPs are closer to their corresponding RNA editing sites, have a larger impact on the number of paired bases, and have a greater effect on the minimum free energy of the predicted RNA secondary structure, compared to control SNPs (Figure 6A, B, C; Park 2017). We also provided examples that SNPs disrupting or creating A:C mismatch within a double stranded Alu hairpin regulate RNA editing, consistent with prior reports that this type of structure provides a favorable target for ADAR (RNA. 2001 7(6):846-58; Nat Struct Mol Biol. 2016 23(5):426-33).

Given that we had previously performed this analysis in our earlier publication, we originally did not repeat this analysis in this present manuscript and instead decided to focus on reporting new findings and biological insights from a variety of new analyses. However, in light of the reviewer’s comment, we have performed this analysis on the GTEx edQTL sites and added three additional panels to an existing supplemental figure (Additional file 7: Figure S4). On this much larger set of edQTL sites, our observations were consistent with our prior findings.

We added the following text to Page 7 of the revised manuscript to describe this new analysis:

**“To comprehensively investigate the effects of edQTLs on computationally predicted RNA secondary structure, we adopted the approach from Park 2017. We found that edQTL SNPs**

**are closer to their corresponding RNA editing sites, have a larger impact on the number of paired bases, and have a greater effect on the minimum free energy of the predicted RNA secondary structure, compared to control SNPs (Additional file 7: Figure S4C, D, E). These results are consistent with our prior observation on a much smaller set of edQTL sites in a single cell type (Park 2017).”**

***Reviewer #2:***

Additional file 7: Figure S4C, D, E

(C) Cumulative distribution plot comparing the absolute value of the distance between SNP-RNA editing site pairs for significant edQTL SNPs and control SNPs within the computationally predicted RNA secondary structure of the IRAlu hairpin.
(D) Cumulative distribution plot comparing the absolute value of the change in the number of paired bases for significant edQTL SNPs and control SNPs.
(E) Cumulative distribution plot comparing the absolute value of the change in free energy of the predicted RNA secondary structure for significant edQTL SNPs and control SNPs. The Kolmogorov-Smirnov test was used for the cumulative distribution plots.

*In the manuscript entitled "Genetic variation and microRNA targeting of A-to-I RNA editing fine tune human tissue transcriptomes", Park et al. identified edQTL events in 49 tissues across 437 individuals. They observed that certain microRNAs are able to differentiate between the edited and unedited isoforms of their targets, and microRNAs can generate eQTL signal from an edQTL locus by microRNA-mediated transcript degradation through the regulation of RNA editing. They also found that some of edQTLs are associated with GWAS signals. These results provide an additional layer of control in the regulation of gene expression and expands our understanding of the functional consequences of RNA editing in human cells. This work is potentially of broad interest to RNA editing, microRNA, QTL, and genetic variants community in that the authors showed a mechanism in which RNA editing variability can influence the phenotypes of complex traits and diseases by altering the stability and steady-state level of critical RNA molecules. This*

*study is novel and interesting, especially in the broad area of genetic architecture. The methods used are solid and the data are also well presented and discussed.*

We thank the reviewer for the highly positive comment of our work. The reviewer considers our study “novel and interesting, especially in the broad area of genetic architecture”.

*Major comments:*

*1. In the miRNA expression analysis, the authors used RNA-seq reads that align to the primary miRNA transcript as a proxy for miRNA measurement of the mature form. It is little concern that if the primary miRNA transcript can reflect mature miRNA expression. It is better for the authors to validate by using independent dataset.*

We agree with the reviewer that it would be preferable to quantify miRNA levels using an independent dataset generated by a method that directly measures mature miRNA levels, rather than using a proxy method that we employed in this work. However, this type of dataset does not exist for our tissue and cell type of interest.

Specifically, in this work we estimated the levels of miR-125a-3p in lymphoblastoid cell lines, cultured fibroblasts, and spinal cord. These three tissues were the only three where an edQTL signal for DHFR was called. However, prior studies that quantified mature miRNA levels in human tissues did not analyze these three tissue types, and GTEx does not have an accompanying dataset of mature miRNA levels. Therefore, we used GTEx poly-A selected RNA-seq reads that align to the cleavage product of the primary miRNA transcript as a proxy for the mature miRNA. We should note that this approach has a precedent in the literature. A similar approach had been used by the FANTOM consortium to estimate mature miRNA levels using CAGE (an approach to measure the 5’ ends of RNA molecules) (Nat Biotechnol. 2017 35(9): 872–878.). The authors were able to measure levels of the 5’ ends of the cleavage product of the primary miRNA from CAGE data and found that these levels were correlated with the levels of the mature miRNAs. On the basis of this observation, the authors used the levels of the 5’ ends of the cleaved primary miRNAs as a proxy for the levels of the mature miRNAs.

To demonstrate the validity of the proxy method we employed in this work, we compared this method as applied to GTEx tissues with a published miRNA qPCR dataset of human tissues (BMC Genomics. 2007; 8: 166), on a set of 20 tissues shared between the two datasets. We compared miRNA quantifications of four well-characterized tissue-specific miRNAs (brain-specific miR-9, brain-specific miR-124a, liver-specific miR-122a, muscle-specific miR-1), as well as miR-125a (the miRNA analyzed in our work by the proxy method). For the four tissue-specific miRNAs, the proxy method performed as well as qPCR in identifying their tissue specificity (Additional file 11: Figure S7A, B, C, D). For miR-125a, the miRNA levels estimated by the proxy method and by qPCR had a Spearman correlation of 0.55 (Additional file 11: Figure S7E), even though the tissue materials and sampling sites were not identical between the two datasets.

Taken together, these data suggest that this proxy approach provides a reasonable approximate estimate of mature miRNA levels, in the absence of data from direct measurements of mature miRNA levels. An alternative would be for us to obtain RNAs from these three tissue types and quantify miR-125a ourselves by qPCR. However, this approach has its own problems, because the source tissues we would obtain from commercial vendors would be different from the GTEx tissues, therefore the quantification would be subject to biological and technical variations and may not accurately reflect mature miRNA levels in GTEx tissues. Additionally, this experiment is practically impossible to do at the moment due to the COVID19 shutdown at our institution.

We added the following text to Page 10-11 of the revised manuscript to provide a brief justification for the proxy method:

Additional file 11: Figure S7. Comparison of miRNA quantification between qPCR and the RNA-seq based pri-miRNA proxy method. Bar plots comparing qPCR and the proxy methods for (A) brain-specific miR-9, (B) brain-specific miR-124a, (C) liver-specific miR-122a, and (D) muscle-specific miR-1. (E) Scatter plot of miR-125a levels estimated by qPCR and the proxy method for a set of tissues shared between the two datasets. The blue line represents a linear fit of the data and the gray region represents the 95% confidence interval.

**“We should note that in these analyses, the expression levels of miR-125a-3p in the three tissues were estimated via a proxy approach, based on GTEx poly-A selected RNA-seq reads that align to the cleavage product of the primary miRNA transcript. This proxy approach has a precedent in the literature. A similar approach has been evaluated and used by the FANTOM consortium to estimate mature miRNA levels using CAGE (an approach to measure the 5’ ends of RNA molecules) (Nat Biotechnol. 2017 35(9):872-878.). To assess the validity of the proxy method we employed in this work, we compared this method as applied to GTEx tissues with a published miRNA qPCR dataset of human tissues (BMC Genomics. 2007 8:166.), on a set of 20 tissues shared between the two datasets. We compared miRNA quantifications of four well-characterized tissue-specific miRNAs (brain-specific miR-9, brain-specific miR-124a, liver-specific miR-122a, muscle-specific miR-1), as well as miR-125a . For the four tissue-specific miRNAs, the proxy method performs as well as qPCR in identifying their tissue specificity (Additional file 11: Figure S7A, B, C, D ). For miR-125a , the miRNA levels estimated by the proxy method and by qPCR had a Spearman correlation of 0.55 (Additional file 11: Figure S7E), even though the tissue materials and sampling sites were not identical between the two datasets. Taken together, these data suggest that this proxy approach provides a reasonable approximate estimate of mature miRNA levels.”**

*2. In line 119-130, the authors found 3,117 unique RNA editing sites that are associated with at least one edQTL SNP across 49 tissues, and showed the number of RNA editing sites in each tissue. Do these RNA editing sites show tissue-specific or shared across tissues? How many edQTL SNPs are associated with RNA editing sites in each tissue? The similar analysis is needed in ASED analysis.*

To address how many edQTL/ASED sites were called in multiple tissues vs called in only one tissue, we created a new supplemental figure (Additional file 6: Figure S3) which extends the data from Figure 2C. Additional file 6: Figure S3A shows the number of shared vs tissue-specific edQTL/ASED sites in each tissue. Additional file 6: Figure S3B shows the proportion of shared vs tissue-specific edQTL/ASED sites in each tissue. Here, an edQTL or ASED site is defined as shared if it is called in more than one tissue, and tissue-specific if it is called in only one tissue. These new analyses show that a large proportion of edQTL/ASED sites are shared across multiple tissues, while certain edQTL/ASED sites are called in a single tissue.

Additional file 6: Figure S3. Stacked bar plots for the number (A) and proportion (B) of shared and tissue-specific edQTL sites (left) and ASED sites (right) across all tissues. Tissues are sorted by the number of edQTL sites.

We added the following text to Page 6 of the revised manuscript to describe this new analysis:

**“Furthermore, edQTL/ASED sites are often shared between tissues (Additional file 6: Figure S3).”**

The reviewer also asked how many edQTL SNPs are associated with RNA editing sites in each tissue. In our work, we have focused our analysis on cis regulated RNA editing sites. Due to the LD structure of the human genome, for each edQTL site we expect to have many significant SNPs in LD with each other, and it is not biologically meaningful to list and count all SNPs that are associated with a given RNA editing site in each tissue.

*Minor comments that may need to be clarified:*

1. *In the simulation of miRNA effects on edQTL and eQTL signal, could the authors explain more clearly why to select a fixed degradation at 20%, 40%, and 80%?*

20%, 40%, and 80% were chosen as a range of degradation rates in our simulation analysis to demonstrate the effect of miRNA-mediated transcript degradation on edQTL and eQTL signals. We can certainly pick other arbitrary values (25%, 50%, 75%), but these would not materially change the observed trend and interpretation.

We added a statement in the Methods section to address this question:

**“These values were chosen to demonstrate the effect of miRNA-mediated transcript degradation on edQTL and eQTL signals across a range of degradation rates.”**

2. *In line 110-111, how to define a variable RNA editing site? Based on the difference of RNA editing level?*

We thank the reviewer for raising this issue. As described in the Methods section, to select variable RNA editing sites, we required “a minimum of 10% difference between the editing levels of the 90% quantile and the 10% quantile across all individuals.” To clarify this issue, we added a reference of the Methods section to this sentence in the Results section, which now reads as:

**“We restricted our analysis to annotated RNA editing sites and applied a set of filters to focus on RNA editing sites that are expressed and variable between individuals (see Methods).”**

3. *In line 392 please explain clearly how to use the linear model to perform edQTL analysis.*

We added the following text to this section to clarify how we used the linear model to perform edQTL analysis:

**“We used the lm function within R to regress the editing level (Φ) against the genotype across individuals of a given tissue in order to obtain a p-value for each SNP.”**

4. *In line 167, "In some tissues, RNA editing is associated with rs2707852." This sentence seems not completed.*

We thank the reviewer for catching this issue and we have modified this sentence to clarify this statement. The updated sentence now reads as:

**“RNA editing is significantly associated with rs2707852 in some tissues but not in others.”**

5. *In figure 2D, 3A, 3E, 4A, 5A, and 6D, please mark how many samples are in each genotype. In the Figure 4A, it seems that the sample size for AA genotype is small.*

For the figures listed, we have updated the figures to include the number of samples for each genotype. Please note that some samples from a given genotype may have been excluded from the analysis and plot because of low RNA-seq coverage of the given RNA editing site (see Methods). For the AA genotype in Figure 4A, as mentioned in the text, these transcripts are preferentially degraded compared to the transcripts in other genotypes. This preferential degradation resulted in lower RNA-seq coverage and a reduced number of samples that passed our filters. We added a note of this on Page 12 of the revised manuscript:

**“This preferential degradation may account for the smaller than expected sample size of the AA genotype in Figure 4A, as certain samples were excluded from the analysis and plot due to low RNA-seq coverage of the RNA editing site.”**

6. *In line 232-233, the authors mentioned that "this suggests a possible genetic mechanism affecting predispositions to cancer", they use the GTEx tissues to quantify the edQTL. How can this linked to cancer?*

We thank the reviewer for raising this point. It is actually entirely possible to link data from GTEx (normal) tissues to cancer, since it is well established that natural variation of gene expression in normal tissues can affect cancer predisposition. In fact, several groups have demonstrated the utility of the GTEx data to identify potential cancer susceptibility genes by identifying genetic variants that are associated with gene expression levels then linking these associations to GWAS signals of cancer (Nat Genet. 2018 50(7): 968–978; Nat Commun. 2018; 9: 4079).

Here, the reviewer was referring to an edQTL in NEIL1 which is associated with a change in the amino acid sequence and altered enzymatic activity of this DNA repair enzyme. When the activity of a DNA repair enzyme is reduced, mutations can accumulate and trigger carcinogenesis. In our analysis, we showed that RNA editing in NEIL1 is associated with genetic variation. By integrating the knowledge that altered RNA editing in NEIL1 may result in altered DNA repair efficiencies, we reasoned that this could result in a genetic predisposition to cancer. We have added text to the manuscript to clarify this point:

**“This suggests a possible genetic mechanism in which altered DNA repair activity could result in an accumulation of mutations and an increased predisposition to cancer.”**

**Second round of review**

**Reviewer 1**

I thank the authors for their detailed reply letter, and the improvements made in the paper. Most of my questions were satisfactorily answered. However, my first point was probably not written clearly enough, and seems to have been misunderstood.

The simplest explanation to the variations observed across tissues is that when editing is generally low (due to low ADAR expression, for example) the *same*allele dependence will be harder to observe. Accordingly, I suggested using a *multiplicative* null model, which means the following:

Phi_ij = (mu_i +alpha Genotype_j) Tissue_dependent_factor

For example, this model will predict the same genotype dependence being manifested as editing levels 10%,20%,30% for AA,AG,GG genotypes in one tissue, but 1%,2%,3% in another weakly-edited tissue. The latter will probably be hard to observe (or to be statistically significant), and this explains the low number of observed edQTLs in muscle. The authors seem to have interpreted the interaction term as an indication against this simple model, but in fact the multiplicative model above includes such a term. In fact, Figure S2 actually shows that the low number of tested sites (by itself, likely a proxy of low editing level) explains the low number of muscle edQTLs.

Indeed, it would be helpful to provide the number of tested sites and the number of tested site-SNP pairs, per tissue.

I have addition clarifying questions regarding the statistics. If I understand correctly, each site passing the initial filters is tested against all >5%-prevalence SNPs within 400kb window passing. Then, the most significant (lowest p) association is picked. Such a procedure, of choosing the best of many p-values, is bound to produce a skewed distribution of p-values even under the null hypothesis. The authors are aware of that, and use a permutation approach to define the FDR=10% cutoffs presented in Table S10.

My questions then are:

(1) Table S10 shows values close to, but generally lower than 1e-5. In many tissues, the cutoff is even lower than 1e-6. Why then is the 1e-5 cutoff used? How many sites will be removed if one uses the actual FDR=10% cutoff?

(2) Figure 2A presents a qq-plot, and asserts that the straight black line represents the expected p-values (presumably meaning, expected under the null hypothesis). Isn’t this line just a y=x line? If so, I would assume the expected p-value to be actually smaller, as they are picked as the best of many trials (for all SNPs in the window). Isn’t it so?

**Reviewer 2**

In my point of view, I think the authors did an excellent job to address my previous concerns.

**Authors Response**

**Point-by-point responses to the reviewers’ comments:**

***Reviewer #1:***

*I thank the authors for their detailed reply letter, and the improvements made in the paper. Most of my questions were satisfactorily answered. However, I am still not convinced that the novelty of the results is sufficient to justify publication in GB. Specifically, the search for edQTLs is a direct extension of the authors' 2017 paper. While the RNA-seq dataset used is indeed much larger, the outcome is not that much different. The older work has identified 1054 RNA editing events associated with cis genetic polymorphisms, compared with 3117 sites here. In fact, many of the individual examples shown in the figures of the present work have been already found in the 2017 paper (e.g. the sites presented in Fig 2D-E, Fig. 3A and Fig. 4A). As expected, the more significant, larger-effect, sites tend to belong to the third that was found using the more limited dataset.*

*The tissue-dependence presented is mostly explained by the expected dependence on the global per-tissue editing level (See Fig 3C that actually shows a rather flat behavior across tissues). Crossing the new list with known GWAS sites resulted in a few anecdotal stories, and the structural analysis does not provide any interesting information beyond what was presented in 2017.*

We thank the reviewer for this extensive review of our manuscript and sharing the detailed comments and suggestions. We appreciate that the reviewer finds the paper improved and most of the questions satisfactorily answered. We welcome the opportunity to address the novelty of this work over our earlier publication, in which we used a population-scale RNA-seq dataset of lymphoblastoid cell lines to identify genetically regulated RNA editing events (PMID: 28754146; GB 2017).

We agree with the reviewer that in terms of edQTL discovery, this new work is an extension of our 2017 publication from a single cell type to the much larger 49-tissue GTEx dataset. However, the advances presented in this new manuscript, including *new results generated in this new round of revision*, go well beyond simply a larger list of edQTL events. This new work includes numerous new analyses that we did not do and could not do in our earlier study. The 2017 publication was primarily focused on cataloging edQTL events. However, at the time of the study, we were unable to provide any clues or suggest concrete molecular mechanisms by which genetically regulated RNA editing events affect gene products and phenotypes. Despite finding putative GWAS associations, we did not know how edQTL events affect gene activity and function.

By contrast, a major focus of this new work is to elucidate the molecular and functional consequences of edQTL events. To the best of our knowledge, this work is the first to perform colocalization analyses of edQTL and eQTL signals. This novel line of investigation allowed us to discover that *cis genetic variants can causally influence RNA editing levels and gene expression levels simultaneously*, suggesting the interplay between edQTL and eQTL signals.

Importantly, in this new round of revision, by taking advantage of the *comprehensive, multi-tissue edQTL dataset*, we were able to:

1) identify tissue-specific edQTL:eQTL colocalization events
2) attribute some of these tissue-specific colocalization events to tissue-specific microRNA levels
3) experimentally confirm editing-specific microRNA:mRNA regulation using 3’-UTR luciferase reporter assays (described in detail below).

*These analyses were not possible in our 2017 Genome Biology paper because it was on a single cell type.* We now experimentally demonstrate that the DHFR example and our proposed model are indeed generalizable to other genes and RNA editing sites. Together, these new data substantially strengthen *the new concept* provided by the current paper: that microRNAs can generate an eQTL signal from an edQTL locus, via microRNA-mediated transcript degradation in an editing-specific manner. Our findings shed light on a *previously unappreciated mechanism for generating eQTLs in human transcriptomes*, and provide novel mechanistic insights into how edQTLs affect gene products and phenotypes. These are important conceptual advances for multiple fields, particularly gene regulation and RNA editing, and have implications for understanding human genetic variation and disease mechanisms.

*The DHFR example (cis-regulated editing in DHFR that affects miR targeting, and eventually leads to an allele-specific expression) is very nice. However, each of the ingredients (edQTL, editing affecting miR targeting, miR suppression RNA expression) is already known, so while it is a beautiful demonstration, I don't think one such example is of sufficient novelty. Had the authors follow up this example and show this mechanism to be a general one, applying to multiple editing events in many genes, this could have been the focus of an interesting paper.*

We thank the reviewer for the positive feedback on the DHFR example. The reviewer commented that each of the ingredients (edQTL, editing affecting miR targeting, miR suppression of RNA expression) is already known. Although differential targeting of the unedited vs edited DHFR site by miR-125a-3p was reported (J Biol Chem. 2017 292:4873-4884), the previous literature could not have anticipated several novel findings from our study. We demonstrated that 1) the RNA editing event in DHFR is genetically controlled; 2) the edQTL signal colocalizes with the eQTL signal of DHFR; and 3) the edQTL:eQTL colocalization occurs in a microRNA concentration-dependent manner. These are all novel results from this work, and we have modified our manuscript to clarify this point. We should emphasize that until now, there were no data showing that these ingredients could come together in a single gene to create an eQTL. Collectively, they form the basis of a compelling hypothesis, which we prove in this paper.

We agree with and appreciate the reviewer’s suggestion to expand on the DHFR example and show its generalizability. Over the last six months, we have expanded our computational analyses and conducted new experiments to demonstrate that the DHFR example and our proposed model are indeed generalizable to other genes and RNA editing sites. Specifically, we performed integrative analyses of edQTL, eQTL, and microRNA expression profiles across diverse tissues, as well as microRNA sequence complementarity to RNA editing sites, to predict additional edQTL-microRNA pairs for which the microRNA may generate an eQTL signal from an edQTL locus in a tissue-specific manner (Figure 7 of the revised manuscript; shown on the next page).

**Figure 7.** Computational discovery and experimental validation of miRNA-mediated tissue-specific edQTL:eQTL colocalization events.

(A) Flowchart of computational analysis. Each edQTL site was required to have at least one tissue with colocalizing edQTL and eQTL signals (PP4 > 0.75), at least one tissue with non-colocalizing edQTL and eQTL signals (PP1 > 0.75), be in the 3’-UTR, and have an editing-specific miRNA which is differentially expressed (fold change > 2) between the colocalizing and non-colocalizing tissues.
(B) Example of an edQTL event in RPL13 with colocalizing edQTL and eQTL signals in the skin (not sun exposed) (left column) and non-colocalizing edQTL and eQTL signals in the cerebellum (right column). Manhattan plots for edQTL (top row), eQTL (middle row), and scatter plots of -log10(p-value) from edQTL and eQTL signals (bottom row) show the presence (left column) or absence (right column) of edQTL:eQTL colocalization. Colocalization posterior probabilities are shown in parentheses (bottom row).
(C) Differential expression of an editing-specific miRNA (miR-26b-5p) targeting the edited version of RPL13. Tissues expressing high levels of the miRNA have colocalizing edQTL and eQTL signals. Tissues expressing low levels of the miRNA do not have colocalizing edQTL and eQTL signals.
(D) Experimental validation of miRNA-mediated edQTL:eQTL colocalization events. Diagram of the 3’-UTR reporter vector using plasmids containing 3’-UTR fragments with the edQTL RNA editing sites (top). Luciferase mRNA levels measured by qPCR of tested 3’-UTR constructs in the presence of editing-specific miRNAs (bottom left). Each barplot displays the mean and SD of three independent experiments. * p<0.05, ** p<0.01. Diagram indicating the editing-specific targeting of miRNAs to unedited or edited 3’-UTR sequences (bottom right). Guanine was used in place of inosine to indicate an edited site.

Specifically, starting from all 3,117 edQTL sites, we performed edQTL:eQTL colocalization analyses tissue-by-tissue, and identified 88 edQTL sites with tissue-specific colocalization (i.e. strong evidence of colocalization in at least one tissue and non-colocalization in at least one tissue). Focusing on 42 such edQTL sites located in annotated 3’-UTRs, we used TargetScan to identify microRNAs that may specifically target the edited or unedited version of the mRNA, and used the GTEx RNA-seq data to discover tissue-specific microRNAs. By intersecting the TargetScan and GTEx results*, we identified 8 unique edQTL sites involving 14 edQTL-microRNA pairs* (Figure 7A and Table S10 of the revised manuscript). One example involving an RNA editing site in RPL13 and miR-26b-5p is illustrated in Figure 7B-C. Using 3’-UTR luciferase reporter assays, we experimentally tested four of these edQTL sites as well as the DHFR site as a positive control*. For all five sites tested, the experimental data show that the microRNA of interest specifically targets the unedited or edited version of the 3’-UTR for transcript degradation, in a manner consistent with our computational prediction based on the edQTL and eQTL signals* (Figure 7D). These data demonstrate that our proposed model generalizes beyond the DHFR example to other genes and RNA editing sites. We have added a new section in Results (**“Direct edQTL-miRNA interactions mediate tissue-specific edQTL:eQTL colocalization events”**) along with a new figure (**Figure 7** of the revised manuscript) to describe these new analyses and results.

We also added the following text to the Discussion section of the revised manuscript to discuss the broader implication of these new analyses and results:

**“We should note that the list of edQTL events that may generate eQTLs events is expected to be substantially larger than the candidate events identified in Figure 7. In our analysis, in order to hone into potential miRNAs computationally, we used stringent criteria to identify tissue-specific edQTL:eQTL colocalization and matched these signals with tissue-specific miRNAs identified from the GTEx data. We expect that numerous miRNAs can also generate eQTLs from edQTLs in a non-tissue-specific manner, although these candidates would be harder to identify computationally. Indeed, the DHFR example could not be identified using the computational strategy and stringent criteria outlined in Figure 7, because all three tissues with significant edQTL signals had high or moderate colocalizing eQTL signals.**

**Our study focused on miRNAs as trans-acting regulators that generate eQTLs from edQTLs; however, we expect that a similar scenario could occur to RNA binding proteins that regulate RNA stability via sequence-specific protein-RNA interactions. In fact, 14% of edQTL sites (443 out of 3,117) have at least one tissue in which the edQTL signal colocalizes with the eQTL signal, suggesting that a trans-acting regulator (miRNA or RNA binding protein) may alter RNA stability in an editing-specific manner. In theory, editing-specific transcript stability control by RNA binding proteins could also generate an eQTL from an existing edQTL. It has been demonstrated that ELAV1/HuR stabilizes the edited version of CTSS transcripts[50]. If ELAV1/HuR preferentially binds and stabilizes the edited or unedited version of an edQTL site, an eQTL signal should emerge. Collectively, our results suggest that RNA editing underlies a previously unappreciated mechanism for generating eQTLs in human transcriptomes, and we provide computational and experimental evidence for the role of miRNAs in creating eQTLs from edQTLs.”**

*My first point was probably not written clearly enough, and seems to have been misunderstood.*

*The simplest explanation to the variations observed across tissues is that when editing is generally low (due to low ADAR expression, for example) the *same*allele dependence will be harder to observe. Accordingly, I suggested using a *multiplicative* null model, which means the following:*

*Phi_ij = (mu_i +alpha Genotype_j) Tissue_dependent_factor*

*For example, this model will predict the same genotype dependence being manifested as editing levels 10%,20%,30% for AA,AG,GG genotypes in one tissue, but 1%,2%,3% in another weakly-edited tissue. The latter will probably be hard to observe (or to be statistically significant), and this explains the low number of observed edQTLs in muscle. The authors seem to have interpreted the interaction term as an indication against this simple model, but in fact the multiplicative model above includes such a term. In fact, Figure S2 actually shows that the low number of tested sites (by itself, likely a proxy of low editing level) explains the low number of muscle edQTLs.*

We appreciate the reviewer’s clarification, and we now understand the point the reviewer tried to make. The initial confusion stems from the fact that we view the genotype effects on RNA editing as the *absolute difference* in RNA editing levels among genotypes, whereas, from our understanding of this comment, the reviewer views them as the *ratio* of RNA editing levels among genotypes. We agree with the reviewer that the observed differences among tissues may be attributed to differences in baseline RNA editing levels, as dependent on the concentrations of RNA editing enzymes in a given tissue.

However, in our view, this interpretation does not minimize the discovery of tissue-specific variations in edQTL signals. Prior to this work, tissue-specific variations of edQTL signals in humans were unknown. Our data suggest that both ADAR and ADARB1 enzymes act in trans in a tissue-specific manner to contribute to variations in edQTL signals across tissues, in particular in skeletal muscle and brain, respectively. Regardless of the underlying molecular interpretation, if we use the hypothetical example provided by the reviewer (or the GSR RNA editing site in Figure 3A) to consider the net effect on transcript products, the outcome remains that a genetic variant affects the editing of a larger percentage (number) of RNA molecules in one tissue, over a second tissue where ADAR (or ADARB1) is lowly expressed. The difference would be 20% vs 2% if we compare the two homozygous genotypes using the reviewer’s hypothetical example. This difference may in turn result in tissue-specific effects of edQTL events on gene activity and function.

We do appreciate the point though, and in light of the reviewer’s comment, we added a note to the revised manuscript to state that the observed tissue-by-tissue variations in edQTLs may be attributed to differences in baseline RNA editing levels, as dependent on the concentrations of RNA editing enzymes in a given tissue.

*Indeed, it would be helpful to provide the number of tested sites and the number of tested site-SNP pairs, per tissue.*

We thank the reviewer for this suggestion. As per the reviewer’s request, we have added an additional supplementary table (Table S11) with the number of tested sites and the number of tested site-SNP pairs per tissue.

*I have addition clarifying questions regarding the statistics. If I understand correctly, each site passing the initial filters is tested against all >5%-prevalence SNPs within 400kb window passing. Then, the most significant (lowest p) association is picked. Such a procedure, of choosing the best of many p-values, is bound to produce a skewed distribution of p-values even under the null hypothesis. The authors are aware of that, and use a permutation approach to define the FDR=10% cutoffs presented in Table S10.*

*My questions then are:*

*(1) Table S10 shows values close to, but generally lower than 1e-5. In many tissues, the cutoff is even lower than 1e-6. Why then is the 1e-5 cutoff used? How many sites will be removed if one uses the actual FDR=10% cutoff?*

We used 1e-5 as the p-value cutoff in this work, to match the same p-value cutoff used by the NHGRI-EBI GWAS Catalog. Although this threshold was more relaxed than FDR of 10%, we decided to adopt

this cutoff for putative edQTL sites, following the same rationale by the GWAS Catalog to include hits with suggestive signals. Using the more stringent FDR≤10% cutoff would, on average, decrease the number of edQTL sites by 30% for each tissue. The total number of unique edQTL sites across all tissues would decrease from 3,117 to 2,087. This would not change the main messages of this manuscript. Additionally, if readers are interested in working with this refined subset of data, they can easily generate this subset by using the p-values corresponding to FDR=10% in each tissue (Table S12) to filter the list of edQTL sites (Table S2).

*(2) Figure 2A presents a qq-plot, and asserts that the straight black line represents the expected p-values (presumably meaning, expected under the null hypothesis). Isn't this line just a y=x line? If so, I would assume the expected p-value to be actually smaller, as they are picked as the best of many trials (for all SNPs in the window). Isn't it so?*

The reviewer is correct that the straight black line in Figure 2A is the y=x line. For the data in the qq-plot, the expected p-values were generated from one permutation, calculated in the same way as how the observed p-values were generated from the real data. The qq-plot is expected to trace the y=x line quite closely for small -log10(p-values), but deviate from the line at large -log10(p-values), indicating statistically significant edQTL sites.

**Third round of Review**

**Reviewer 1**

I find the new version of the manuscript very interesting. Specifically, it presents clear support for the new model, where edQTLs lead to eQTLs through miRNA re-targeting. I thus recommend publication (after the authors consider my questions below), but think that these new data should be the focus of the paper. I maintain my position that the increase in the number of edQTLs (2087 statistically significant sites, at FDR<=10%, compared to 1054 in a previous publication), and the tissue-dependence of detected edQTLs (mainly reflecting ADAR expression, and therefore quite expected) are not of much significance. In my opinion the paper would be much improved if these parts (p.7 to p.11) would be shortened substantially, so that it is clear to the reader that the main goal of this work is to present a molecular mechanism for the edQTL-eQTL correlation (possibly one out of many, see below).

For example, the following structure may be considered:

(i)	A short description of the edQTL detection, which is basically a straight forward extension of earlier work. Much of Fig. 1,2,3 could be removed or transferred to Supp. Info as these figures present ideas shown in the earlier work, or (in the case of tissue-specificity) results that are more or less expected based on ADAR activity profile. (ii) Presenting the DHFR example as a trigger for hypothesizing the model (iii) A systematic search (the new computational pipeline of Fig. 7) of colocalization and correlation with miR profiles, and experimental validation.

With this in mind, I have a few questions and comments.

Main points:

1. Unlike the case of colocalization of polygenic traits, edQTLs are expected to be in close vicinity of the edited site (could you provide a distribution of the genomic distance for the 2087 edQTLs?), and many eQTLS are expected to be within the transcript. Thus, one may argue that colocalization of edQTL and eQTL within a window of tens of kbs is not a very strong support of a connection. Please discuss this point.

2. Related to the previous point: how common is it to find an eQTL within (unedited) 3’UTRs? This might help assess the strength of the evidence of having the eQTL near the edQTL in the UTR.

3. The authors report 443 sites that colocalize with the expression level. The newly introduced pipeline results in only a few of these (8 sites in Table S10) that could be explained by the miR retargeting model. Do you believe this mechanism to be much more prevalent than the 8 sites found, and explain a large fraction of the 443 sites? Are there other possible mechanisms in play?

4. The current pipeline employs tissue specificity to identify edQTL-miRNA-eQTL interactions. This is a very good technique to minimize detection noise. However, in principle one can imagine the same model working (for some other sites) across tissues. Can you try and identify such cases, or comment on this possibility?

Minor issues:

1. I understand why the authors would like to maintain “suggestive” sites even if they are not statistically significant, but I don’t think it’s a good practice to report them and rely on the reader to find the significance cutoff in a separate table. Please add to Table S2 the FDR-adjusted p-value, in addition to the uncorrected one.

2. (Related to Major Point #1) How is the significance of the edQTL-eQTL localization assessed? Does the posterior probability take into account the fact that the edQTL SNP is usually very close to the editing site?

**Authors Response**

**Point-by-point responses to the reviewers’ comments:**

**Reviewer #1:**

I find the new version of the manuscript very interesting. Specifically, it presents clear support for the new model, where edQTLs lead to eQTLs through miRNA re-targeting. I thus recommend publication (after the authors consider my questions below), but think that these new data should be the focus of the paper. I maintain my position that the increase in the number of edQTLs (2087 statistically significant sites, at FDR<=10%, compared to 1054 in a previous publication), and the tissue-dependence of detected edQTLs (mainly reflecting ADAR expression, and therefore quite expected) are not of much significance. In my opinion the paper would be much improved if these parts (p.7 to p.11) would be shortened substantially, so that it is clear to the reader that the main goal of this work is to present a molecular mechanism for the edQTL-eQTL correlation (possibly one out of many, see below).

For example, the following structure may be considered:

(i) A short description of the edQTL detection, which is basically a straight forward extension of earlier work. Much of Fig. 1,2,3 could be removed or transferred to Supp. Info as these figures present ideas shown in the earlier work, or (in the case of tissue-specificity) results that are more or less expected based on ADAR activity profile. (ii) Presenting the DHFR example as a trigger for hypothesizing the model (iii) A systematic search (the new computational pipeline of Fig. 7) of colocalization and correlation with miR profiles, and experimental validation.

With this in mind, I have a few questions and comments.

We thank the reviewer for the positive comment about our revised manuscript, especially the new data we presented. The reviewer suggested us to substantially shorten the section on edQTL/ASED analyses and move the results to supplementary materials. In our view, the comprehensive edQTL/ASED results on the large-scale GTEx data, although not methodologically novel, provide a rich resource to the community and reaches a broader audience of scientists studying diverse tissues and diseases. After communicating and consulting with our handling editor at Genome Biology, we have decided to leave the manuscript structure as-is.

Main points:

1. Unlike the case of colocalization of polygenic traits, edQTLs are expected to be in close vicinity of the edited site (could you provide a distribution of the genomic distance for the 2087 edQTLs?), and many eQTLS are expected to be within the transcript. Thus, one may argue that colocalization of edQTL and eQTL within a window of tens of kbs is not a very strong support of a connection. Please discuss this point.

The colocalization analysis is not based on the genomic distance between two QTL signals (e.g. edQTL and eQTL). Rather, given two traits of interest (RNA editing level and gene expression level), the colocalization analysis examines and compares the overall distribution of association p-values for all SNPs in a large genomic window (400 kb in our study). Intuitively, a high posterior probability of colocalization (PP4 for the coloc software) is reached if the two sets of p-value distributions track each other. On the other hand, it is also common that the QTL signals of two distinct traits are in the vicinity of each other in terms of their genomic loci, but their p-value distributions do not track. This will result in a low posterior probability of colocalization, indicating that the two traits (e.g. edQTL and eQTL) do not share the same underlying causal variant.

To clarify this issue, we added the following text to Page 20 of the revised manuscript:

**“We should note that the colocalization analysis is not based on the genomic distance between two QTL signals (e.g. edQTL and eQTL). Rather, given two traits of interest (RNA editing level and gene expression level), the colocalization analysis examines and compares the overall distribution of association p-values for all SNPs in a large genomic window. Intuitively, a high posterior probability of colocalization is reached if the two sets of p-value distributions track each other.”**

2. Related to the previous point: how common is it to find an eQTL within (unedited) 3'UTRs? This might help assess the strength of the evidence of having the eQTL near the edQTL in the UTR.

This question is related to the reviewer’s previous point, and we have clarified in our response above that the colocalization analysis is not based on the genomic distance between two QTL signals.

3. The authors report 443 sites that colocalize with the expression level. The newly introduced pipeline results in only a few of these (8 sites in Table S10) that could be explained by the miR retargeting model. Do you believe this mechanism to be much more prevalent than the 8 sites found, and explain a large fraction of the 443 sites? Are there other possible mechanisms in play?
4. The current pipeline employs tissue specificity to identify edQTL-miRNA-eQTL interactions. This is a very good technique to minimize detection noise. However, in principle one can imagine the same model working (for some other sites) across tissues. Can you try and identify such cases, or comment on this possibility?

These are excellent points. In fact, we had discussed these issues in the last revision (page 22-23 of the current manuscript). Specifically, we noted that “**the list of edQTL events that may generate eQTLs events is expected to be substantially larger than the candidate events identified in Figure 7**”, and that “**numerous miRNAs can also generate eQTLs from edQTLs in a non-tissue-specific manner, although these candidates would be harder to identify computationally**”. We also noted that “**editing-specific transcript stability control by RNA binding proteins could also generate an eQTL from an existing edQTL**”. Given the already extensive discussions (two full paragraphs on page 22-23), we do not plan to add more text to discuss these issues. We agree with the reviewer that it would be interesting to find additional cases and mechanisms for generating eQTLs from edQTLs, but we believe that this is beyond the scope of this work.

Minor issues:

1. I understand why the authors would like to maintain "suggestive" sites even if they are not statistically significant, but I don't think it's a good practice to report them and rely on the reader to find the significance cutoff in a separate table. Please add to Table S2 the FDR-adjusted p-value, in addition to the uncorrected one.

In our study, we used a permutation-based approach to estimate the nominal p-value corresponding to the 10% FDR for each tissue. We have not calculated FDR-adjusted p-values for all nominal p-values. To address the reviewer’s comment, we have added a new column to Table S2 to indicate whether an edQTL event passes the 10% FDR threshold in a given tissue.

2. (Related to Major Point #1) How is the significance of the edQTL-eQTL localization assessed? Does the posterior probability take into account the fact that the edQTL SNP is usually very close to the editing site?

The edQTL-eQTL colocalization is assessed with the coloc software package, which is widely used for colocalization analysis (PLoS Genet. 2014 10(5):e1004383). To define colocalization, we required the posterior probability PP4 to be greater than 0.75, which is a commonly used threshold for the coloc software. As we have clarified in response to the reviewer’s main point #1 above, the colocalization analysis is not based on the genomic distance between two QTL signals. Rather, given two traits of interest (RNA editing level and gene expression level), the colocalization analysis examines and compares the overall distribution of association p-values for all SNPs in a large genomic window, to determine if the two sets of p-value distributions track each other.
